# *Leishmania infantum* 5’-Methylthioadenosine Phosphorylase presents relevant structural divergence to constitute a potential drug target

**DOI:** 10.1186/s12900-017-0079-7

**Published:** 2017-12-19

**Authors:** Hela Abid, Emna Harigua-Souiai, Thouraya Mejri, Mourad Barhoumi, Ikram Guizani

**Affiliations:** 1 Laboratory of Molecular Epidemiology and Experimental Pathology (LR11IPT04/ LR16IPT04), Institut Pasteur de Tunis, Université de Tunis El Manar, Tunis, Tunisia; 2 Faculté des Sciences de Bizerte, Université de Carthage, Tunis, Tunisie

**Keywords:** *Leishmania*, MTAP, Homology modeling, Molecular docking, Antibody

## Abstract

**Background:**

The 5′-methylthioadenosine phosphorylase (MTAP), an enzyme involved in purine and polyamine metabolism and in the methionine salvage pathway, is considered as a potential drug target against cancer and trypanosomiasis. In fact, *Trypanosoma* and *Leishmania* parasites lack de novo purine pathways and rely on purine salvage pathways to meet their requirements. Herein, we propose the first comprehensive bioinformatic and structural characterization of the putative *Leishmania infantum* MTAP (*Li*MTAP), using a comparative computational approach.

**Results:**

Sequence analysis showed that *Li*MTAP shared higher identity rates with the *Trypanosoma brucei* (*Tb*MTAP) and the human (*hu*MTAP) homologs as compared to the human purine nucleoside phosphorylase (*hu*PNP). Motifs search using MEME identified more common patterns and higher relatedness of the parasite proteins to the *hu*MTAP than to the *hu*PNP. The 3D structures of *Li*MTAP and *Tb*MTAP were predicted by homology modeling and compared to the crystal structure of the *hu*MTAP. These models presented conserved secondary structures compared to the *hu*MTAP, with a similar topology corresponding to the Rossmann fold. This confirmed that both *Li*MTAP and *Tb*MTAP are members of the NP-I family. In comparison to the *hu*MTAP, the 3D model of *Li*MTAP showed an additional α-helix, at the C terminal extremity. One peptide located in this specific region was used to generate a specific antibody to *Li*MTAP. In comparison with the active site (AS) of *hu*MTAP, the parasite ASs presented significant differences in the shape and the electrostatic potentials (EPs). Molecular docking of 5′-methylthioadenosine (MTA) and 5′-hydroxyethylthio-adenosine (HETA) on the ASs on the three proteins predicted differential binding modes and interactions when comparing the parasite proteins to the human orthologue.

**Conclusions:**

This study highlighted significant structural peculiarities, corresponding to functionally relevant sequence divergence in *Li*MTAP, making of it a potential drug target against *Leishmania*.

**Electronic supplementary material:**

The online version of this article (doi: 10.1186/s12900-017-0079-7) contains supplementary material, which is available to authorized users.

## Background

Neglected Tropical Diseases (NTDs) correspond to multiple transmissible pathologies that mainly occur in tropical and sub-tropical regions. They affect populations living in poverty with more than a billion people in 149 countries worldwide [1]. Here, we focus on leishmaniases, a group of vector-borne diseases caused by different species of protozoan parasites of the genus *Leishmania* [2]. Three hundred and 50 million people are at risk of infection and 2 million cases are reported worldwide each year [3]. One to 1.5 million cases of cutaneous leishmaniasis (CL) and 0.2–0.5 million cases of visceral leishmaniasis (VL) are reported annually [3]. VL is mainly caused by *Leishmania donovani* and *Leishmania infantum* (*L. infantum*) species, with an annual death toll of 50,000 cases [3]. Mainstay therapy is based on the use of toxic pentavalent antimonials in long treatment courses [4]. Furthermore, their prolonged use is increasingly inducing parasite drug resistance [5]. Second line drugs, such as pentamidine, miltefosine, and amphotericin B also are toxic, costly or have adverse effects [6]. Therefore, the need for new targets and new drugs is increasingly important, and constitutes research priority.

Search of novel potential drug targets mainly focuses on biochemical and metabolic pathways that show differences between pathogens and their host. Purine salvage, polyamine biosynthesis and thiol metabolism are among the most important metabolic pathways being considered for drug development against diseases caused by *Trypanosomatidae* parasites [7, 8]. Some of the most striking differences between parasites and their mammalian host are found in purine metabolism [9]. In mammals, the de novo and/or the so-called “salvage” pathways ensure the synthesis of the purine nucleotides. To the contrary, most parasites studied rely on the salvage pathways for their purine requirement as they lack the pathways for de novo purine biosynthesis [9]. Therefore, salvage purine metabolism constitutes potentially an excellent target for the rational design of antiparasitic drugs. Among the enzymes involved in purine metabolism, 5′-methylthioadenosine phosphorylase (MTAP) plays a crucial role in purine and polyamine metabolism and in the methionine salvage pathway [10]. The 5′-methylthioadenosine (MTA), natural substrate of MTAPs, is generated during polyamine biosynthesis and is then cleaved to adenine and 5′-methylthioribose-1-phosphate [10, 11], which are respectively incorporated into the salvage pathways of purine and methionine [12]. MTAP, an entry enzyme to methionine salvage pathway, plays an important role to maintain low intracellular levels of MTA, thus to preserve a proper cellular function. Methionine synthesis, polyamine synthesis, protein trans-methylation and trans-sulfuration pathways are excellent targets for chemotherapeutic intervention against African trypanosomes, which are phylogenetically close to *Leishmania* parasites [13]. MTAP was described as an interesting chemotherapeutic target in African trypanosomes (*Trypanosoma brucei brucei*), for which selective transition-state analogues were developed. We cite the 5′-hydroxyethylthio-adenosine (HETA), an analogue of MTA, which is highly metabolized by the Trypanosome MTAP in comparison to the mammalian enzyme [10, 14]. Growth inhibition assays showed IC_50_ values ≤1 μM for HETA, which was selected among possible candidates for in vivo evaluations. HETA exhibited 70% to 90% cure rates when administered to mice infected with *T. brucei brucei* [10, 14]. Moreover, *Leishmania major* MTAP (*Lm*MTAP) and *Trypanosoma brucei* MTAP (*Tb*MTAP) have high druggability indexes (0.8, range: 0 to 1) according to the TDR (Tropical Disease Research) Targets Database (www.tdrtargets.org). This explains our interest to such proteins as promising drug targets against diseases caused by *Trypanosomatidae* parasites. However so far, no study targeted MTAP in *Leishmania*.

The enzymes that catalyze the phosphorolytic cleavage of the glycosidic bond in nucleosides are structurally classified in two families called nucleoside phosphorylase-I (NP-I) and nucleoside phosphorylase-II (NP-II) [15]. Members of NP-II family share a common two-domain subunit fold and a dimeric quaternary structure while members of the NP-I family, including MTAP, share a characteristic subunit topology with a trimeric or a hexameric quaternary structure. They accept a range of purine or pyrimidine nucleosides as substrates. Multiple MTAP proteins from archaeal, bacterial and mammalian species were characterized on the enzymatic or structural levels [16–24]. It was reported that MTAP functions as a dimer in *Mycobacterium smegmatis* and *Mycobacterium tuberculosis* [21, 22], as a trimer in human MTAP (*hu*MTAP) [18], in *Schistosoma mansoni* (*Sm*MTAP) [24] and in a putative bacterial MTAP (PDBids: 4GLF and 4GLJ) [25], and as a hexamer in *Sulfolobus solfataricus* and *Pyrococcus furiosus* [19, 26]. The first structure of *hu*MTAP was solved at 1.7 Ǻ (PDBid: 1CG6) and the enzyme showed a trimeric quaternary structure very similar to that seen in mammalian purine nucleoside phosphorylase (*hu*PNP) [18]. Since then, other human and bacterial MTAPs were crystallized and deposited in the protein database (www.pdb.org). Despite the existence of multiple quaternary structures within the NP-I family members, the subunit fold is highly conserved [15]. It consists of central β-sheets that form a distorted β-barrel, surrounded by several α-helices, characteristic of the Rossmann fold topology mainly found in proteins that bind nucleotides [15].

The active site (AS) in NP-I family members consists of adjacent phosphate- and nucleoside-binding sites (BSs), mainly constituted by residues from the central β-sheets and the interconnecting loops from one subunit, and residues from an adjacent subunit [15]. While the phosphorolysis reaction is common to all enzymes of the NP-I family, there are significant differences in nucleoside specificity within the family members [15, 27].

Characterizing the AS of an enzyme is a key step towards the identification of novel and selective inhibitors that may constitute lead molecules. It is further important to understand and elucidate the binding mode of the enzyme substrate. Such knowledge is valuable for the design of transition-state analogues, which appear to be interesting inhibitors in the case of NP-I family members [28–35]. Indeed the co-crystal structure of the *hu*PNP with acyclovir diphosphate led to the design of a series of 9-substituted 9-deazapurine analogues that showed IC_50_ values ranging from 17 to 120 nM [28]. A comparative analysis of the binding mode of MTA versus Methylthio-immucillin-A (MTM), a tight-binding transition state inhibitor of *hu*MTAP, onto the AS of *hu*MTAP revealed differential interactions between the natural substrate and its analogous inhibitor [34].

In this context, we aimed to proceed to a comprehensive comparative bioinformatics and structural characterization of the putative *L. infantum* MTAP (*Li*MTAP). Primary sequence alignment (PSA), active site prediction on the PSA, and MEME modeling confirmed closer relationships of *Li*MTAP and *Tb*MTAP, its orthologue in *T. brucei*, to *hu*MTAP than to *hu*PNP. This was further confirmed by EC number predictions using the 3D homology model, thus confirming the annotation of the parasite proteins as 5'-methylthioadenosine phosphorylases. The comparison of the 3D homology models of *Li*MTAP and *Tb*MTAP proteins to *hu*MTAP was thus conducted showing a global conservation of the 2D topology and 3D structure with notable peculiarities like the occurrence of an extra α helix that brings the N- and C- termini to a close proximity, and strikingly different solvent accessible surface areas (SASAs) and electrostatic potentials (EPs) distributions. Different potential binding sites (BSs) were also identified by SOM-BSfinder, save for a cavity corresponding to the active site (AS) that presented a different shape and a larger size on the parasite proteins. Thus, characterization of the binding mode of MTA and HETA into the AS and docking simulations were performed predicting different binding modes and molecular interactions within the active site cavity. In spite of the relative conservation of the AS residues, in line with different EPs and binding modes, these residues interact differently with MTA in the predicted structural alignments. Such differential interactions also explained the selective inhibition of *Tb*MTAP by HETA. Structural differences between *Li*MTAP and *hu*MTAP were exploited to produce a polyclonal antibody that is specific to *Li*MTAP.

## Methods

### Primary sequence alignment and motifs identification

MTAP primary sequences of *L. infantum* (LinJ05.0830) and *T. brucei* (Tb927.7.704) were extracted from the TriTryp database (TriTrypDB; http://www.tritrypdb.org/tritrypdb), where they were annotated as putative MTAPs. The *hu*MTAP and *hu*PNP sequences were extracted from the UniProt Knowledgebase (UniProtKB; http://www.uniprot.org/uniprot), using the accession numbers Q13126 and P00491, respectively. Sequences were aligned under T-Coffee [36] using the clustalw_msa method. Motifs search was performed using MEME [37]. The program was asked to generate eight motifs having 6–50 residues size.

### Generation of the 2D and 3D structures

The secondary structures of *Li*MTAP, *Tb*MTAP and *hu*MTAP (PDBid: 1CG6) were detected using the STRIDE program [38] under the Pro-origami web server **(** http://munk.csse.unimelb.edu.au/pro-origami/; [39]). Advanced options were used to exclude 3_10_ helices and pi helices. Topology diagrams were then generated and manually labeled in order to indicate residue numbers for each secondary structure element.

The 3D structure modeling of the parasite MTAP proteins was performed through I-TASSER server [40, 41] (http://zhanglab.ccmb.med.umich.edu/I-TASSER). Five models were generated for each submitted protein sequence, ranked according to their C-scores as an estimate of their quality [42]. The model with the best (highest) C-score was retained and further refined through the ModRefiner server [43]. Predictions on functional annotations of each protein based on proteins structurally related to the predicted 3D model were provided by I-TASSER. This included Enzyme Commission (EC) numbers and Gene Ontology (GO) terms predictions using COACH [44].

### Surface mapping and active site predictions

The electrostatic potential (EP) on protein surfaces was calculated using the Poisson-Boltzman equation using the Adaptive Poisson-Boltzmann Solver plug-in (APBS) implemented in PyMOL (The PyMOL Molecular Graphics System, Version 1.8 Schrödinger, LLC). Calculations were performed on the refined version of I-TASSER 3D models of *Li*MTAP and *Tb*MTAP, and on the crystal structure of the *hu*MTAP (PDB ID: 1CG6).

An adapted version of SOM-BSfinder [45], a Self-Organizing Maps-based algorithm [46], was used to identify potential binding sites (BSs) on the parasite proteins. The Enamine Golden Fragments (EGF) collection, containing chemically diverse fragments satisfying the Rule of Three [47] was used as probe library. A 3D Self-Organized Map (SOM) of the atomic coordinates of the docked probes and a Unified Distance Matrix (U-matrix) were generated as previously described [45]. Interaction hot spots revealed through the presence of areas with high neuron densities associated with low values in the U-matrix (U-values) were considered as “high neuron consensus”- areas. Regions with low densities associated with high U-value were considered as barriers separating the docking hot spots. We defined a cutoff (t_U_) on the U-values to distinguish potential BSs (consensual binding regions with U-values ≤ t_U_) from barriers between BSs (regions with U-values > t_U_) as: *t*_*u*_ = *m*_*u*_ + √ *v*, where m_U_ and v are respectively the mean and the variance of the U-values.

### Molecular docking

MTA, the natural substrate of MTAP and HETA, an inhibitor of the *Tb*MTAP (see Additional file 1: Figure S1), were docked targeting the AS of the *hu*MTAP PDB entry 1CG6 and the two parasite refined models for *Li*MTAP and *Tb*MTAP, obtained from I-TASSER server. Structure data file (SDF) of the MTA and HETA molecules were downloaded from the PubChem database (https://pubchem.ncbi.nlm.nih.gov/) under the accession numbers 439,176 and CHEMBL191917, respectively. Ligands SDF files and receptors PDB files were converted into the PDBQT format using the Open Babel package [48], as follows: (i) hydrogen atoms were added, (ii) 3D atomic coordinates were generated and (iii) Gasteiger atomic partial charges were calculated. Docking calculations were performed using AutoDock Vina 1.1.2 [49] with its default parameters and up to 20 docking poses for each ligand were asked to be generated. Pairwise atomic Euclidean distance was calculated between each protein and MTA or HETA in their best docking pose, defined as the lowest-energy pose according to the scoring function implemented in AutoDock Vina [49]. Distances lower than or equal to a cutoff of 4Ǻ were considered to determine residues involved in protein-ligand interactions. All interactions were examined and irrelevant ones were removed.

### Cell extracts preparation

*Leishmania infantum* LV50 (MHOM/TN/94/LV50) parasites were cultivated in standard RPMI 1640 Medium supplemented with 2 μM L-glutamine, 1 U/mL penicillin, 0.5 U/mL streptomycin (Gibco BRL, Germany) and 10% heat-inactivated fetal calf serum (FCS, Dutscher, France) at 22 °C. Parasites were collected when cultures reached the stationary phase and were then centrifuged at 1600 g for 20 min. The washed dry pellets were stored at −80 °C until use.

To extract endogenous proteins, frozen parasite pellets, kept on ice, were resuspended in 1 mL of lysis buffer (50 mM Tris-HCl (pH 7.4), 0.1 mM disodium EDTA), containing 0.05 mM of Phenylmethanesulfonyl fluoride (PMSF) as inhibitor of proteases. The cells were sonicated (4 × 10 s) to reduce viscosity and were then centrifuged for 15 min at 1600 g, at 4 °C. The supernatants were dialyzed during 2 h against a buffer containing 50 mM Tris-HCl (pH 7.4), 0.1 mM disodium EDTA, at 4 °C, to eliminate the endogenous phosphate as described previously [35].

Peripheral Blood Mononuclear PBMCs were prepared from heparinized blood, collected from one consented healthy donor (who provided a written consent). The study protocol was approved by the local ethical comittee of the Institut Pasteur de Tunis. The PBMCs were collected by density centrifugation through Lymphocyte Separation medium (Eurobio, France). PBMC were washed two times in 10 ml (1×) PBS at 500 g for 10 min and lysed on ice by sonication (2 × 10 s), in presence of 0.05 mM of PMSF.

Protein concentrations of LV50 and PBMC lysates were determined by the Bicinchoninic acid (BCA) protein assay kit (Sigma, Germany) with bovine serum albumin (BSA) as a standard.

### Western blot

Four putative antigenic *Li*MTAP peptides were predicted using Antigenic, a method described previously [50], implemented in EMBOSS. The peptide (AIVTKPEHIPAETKQRIAPLVASK), located in the C-terminus of *Li*MTAP, was used to produce a polyclonal antibody (Genescript, USA). The specificity of this anti-*Li*MTAP was assessed by western blot.

PBMC lysates were used as human control in western blot. Three amounts (3 μg, 7 μg and 15 μg) of LV50 lysates and 15 μg of PBMC lysates were resolved by electrophoresis on SDS-polyacrylamide gels (12%), transferred onto a polyvinylidene difluoride (PVDF) blotting membrane (GE Healthcare life sciences, Amersham, Germany) and probed by immunoblotting with anti-*Li*MTAP (d: 1/10000) (Genescript, USA) and anti-horseradish peroxidase (HRP) conjugated secondary (d: 1/5000) antibodies (Promega, Madisson WI). The proteins were visualized by the enhanced chemiluminescence detection system (ECL, Pierce, Rockford, IL). The same experiment was assessed using an anti-human β-actin (d: 1/5000) (Cell Signaling Technology, Danvers, MA) as a positive control for human PBMC.

## Results

### Closer relationship of the parasite proteins to the *hu*MTAP and common motif patterns

**Fig. 1:**
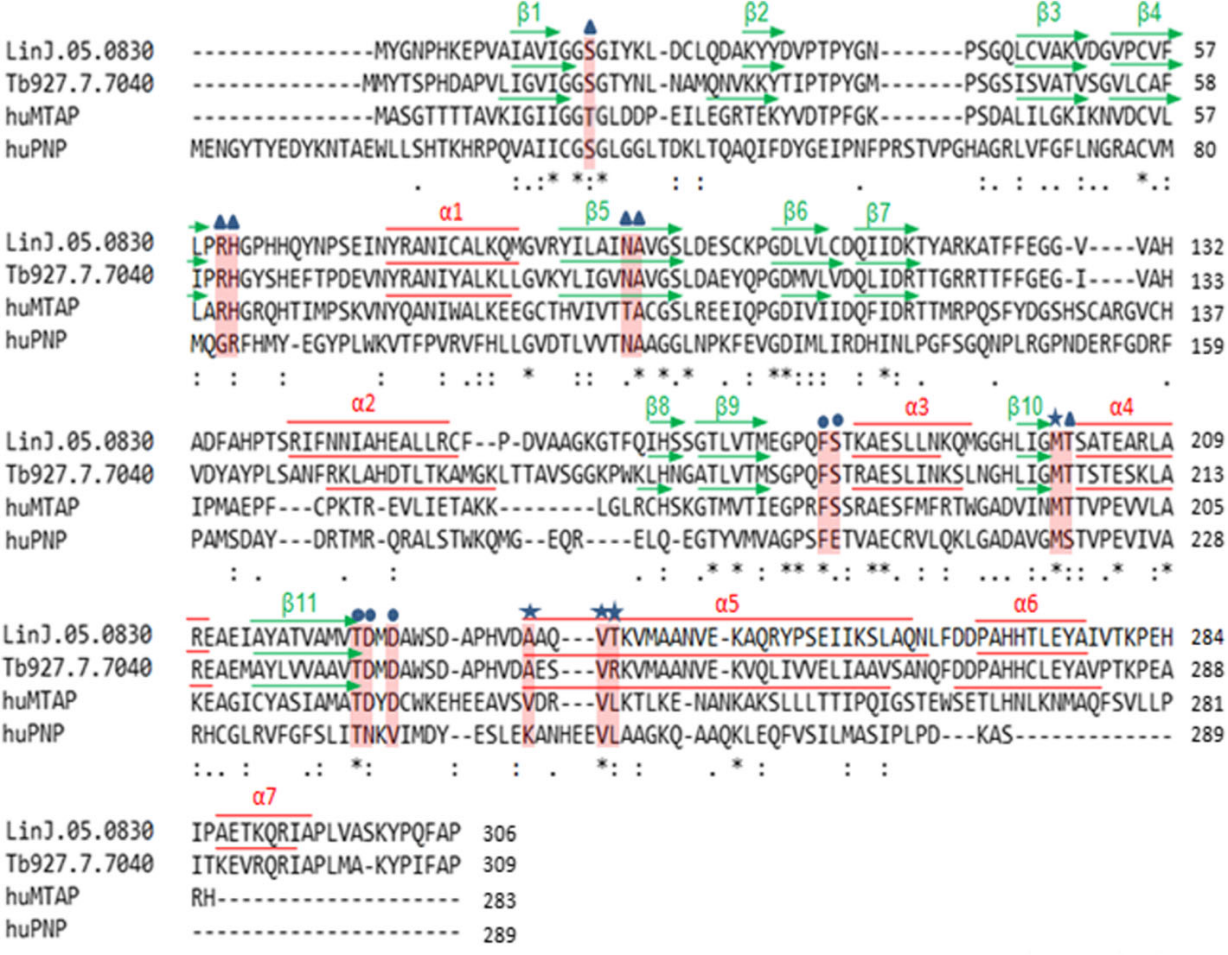
Primary sequence alignment (PSA) of *Li*MTAP, *Tb*MTAP, *hu*MTAP and *hu*PNP obtained using T-coffee program. The PSA indicates regions of sequence divergence relating to the *hu*PNP or to some regions in the N and C termini of the parasite proteins and in their central part. Pink- shaded residues indicate active site residues of the *hu*MTAP and their corresponding residues on *Li*MTAP, *Tb*MTAP and *hu*PNP, on the alignment. These residues were described in human MTAP crystals as base-binding sites (cercles), methylthioribose-binding sites (stars) or sulfate/phosphate-binding sites (triangles). The parasite proteins and *hu*MTAP share 11 out of 15 residues. The secondary structures (β-strands in green arrows and α-helices in red lines) are shown above the parasite proteins and *hu*MTAP sequences. They align well to each other confirming correct PSA alignment

Primary sequence alignment (PSA) of the two parasite proteins, *hu*MTAP and *hu*PNP, was performed using T-Coffee program (Fig. 1). The PSA revealed high divergence on the N- and C-termini of the *hu*PNP compared to the three remaining proteins, along with equivalent identity rates (22–24%). The parasite proteins showed higher identity with the *hu*MTAP (37% and 35%, respectively) than with the *hu*PNP. The *Leishmania* protein has 60% identity with the Trypanosome protein indicating a higher relatedness between these parasite proteins than with the human ones.

Residues involved in the active site (AS) of the *hu*MTAP [15] were shaded in pink on the PSA (Fig. 1). Among these 15 residues, five were conserved on the alignment among the four studied sequences and corresponded to A94, F177, M196, T219 and V236 in *hu*MTAP. Six residues were conserved in the three MTAPs (R60, H61, S178, T197, D220 and D222 in *hu*MTAP). The remaining four residues (T18, T93, V233 and L237) appeared to be specific to the AS of *hu*MTAP. Residues T18 and T93 were replaced by a serine (S) and an asparagine (N) in all three remaining sequences, respectively. Residues V233 and L237 were different from their counterparts in the parasite MTAPs and the *hu*PNP. The V233 was replaced by an alanine in both *Li*MTAP and *Tb*MTAP (A236 and A240, respectively) and by K254 in the *hu*PNP. The L237 was conserved in the *hu*PNP (L261) but replaced by T240 in *Li*MTAP and R244 in *Tb*MTAP.

**Fig. 2:**
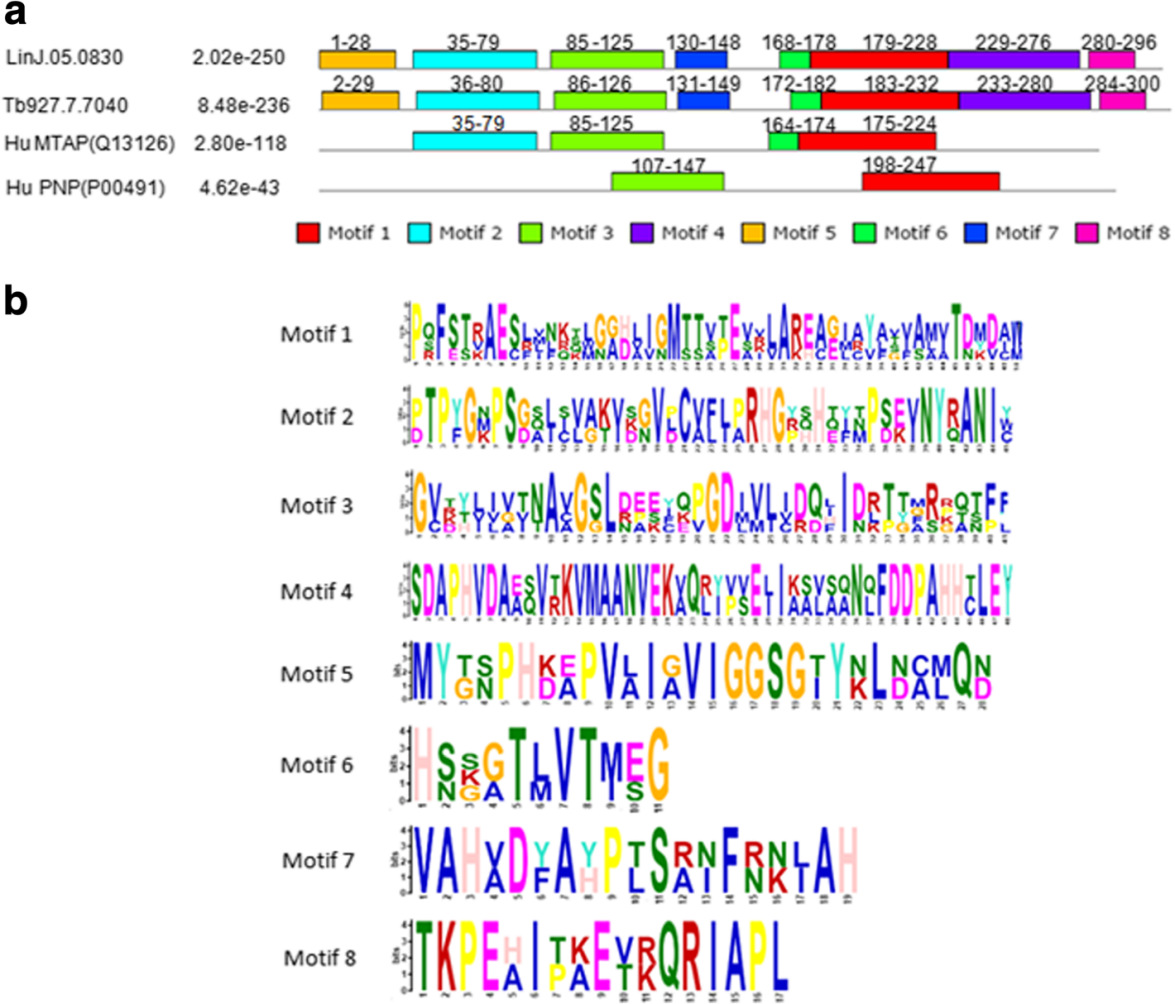
MEME models of the primary sequences of *Li*MTAP, *Tb*MTAP, *hu*MTAP and *hu*PNP. **a** MEME generated primary sequence models made of up to 8 motifs on the 4 proteins. The location of these motifs, represented as color-coded boxes, is shown on a graphical illustration of the proteins primary sequence. Numbers above these boxes indicate the first and last amino acids of each MEME motif. The *p*-value of each MEME modeled protein is indicated. **b** Logo representation of the sequence composition of the MEME motifs in amino acids

Further analysis of the primary sequences was achieved, on the four protein sequences, through motifs identification using MEME. Eight motifs were asked to be found (Fig. 2), as this is the optimal number of characterized motifs found in PNPs and/or MTAPs [15]. Only two motifs were obtained for the *hu*PNP while four to eight motifs were identified for the *hu*MTAP and the parasite proteins. Interestingly, our analysis identified motifs that embedded many motifs previously described as common and characteristic of members of the NP-I family, and more importantly, they covered all the motifs common to the MTAP proteins [15]. The M1 and M3 motifs were conserved in all 4 proteins (Fig. 2a). M1 included smaller motifs previously described as common to trimeric PNPs and MTAPs. However, at its C-terminal part (AA 207–224 in *hu*MTAP), M1 integrated a motif that was previously defined as specific to MTAP proteins [15]. M3 corresponded to a motif that can be found in all members of the NP-I family [15].

Two motifs, M2 and M6, were conserved among the parasite proteins and *hu*MTAP (Fig. 2a). In fact, M2 motif corresponded to a motif already described as specific to MTAP proteins, while M6 motif was not described by Pugmire & Ealick [15]. This could be explained by the small subset of proteins studied herein.

Four motifs M4, M5, M7 and M8 that mapped on variable regions on the PSA (Fig. 1) were specific to the parasite proteins (Fig. 2a). Interestingly, M5 motif contained a sequence (IGIIGGTGL in *hu*MTAP) previously described as a common motif to trimeric PNPs and MTAPs [15] but presented divergent C- and N-termini as compared to the *hu*MTAP. In spite of this common sequence (IGIIGGTGL in *hu*MTAP), the program identified M5 motif as specific to the parasite proteins. Motif 7 was located in the central region (AA 130–148 on *Li*MTAP) and M4 and M8 motifs covered the C-terminal region on *Li*MTAP and *Tb*MTAP (Fig. 2a). Overall, the motifs that were specific to the parasite proteins (M4, M5, M7 and M8) had more sequence conservation among the two parasite proteins respectively than the remaining motifs (Fig. 2b). The M8 motif presented the most conserved sequence (Fig. 2). Blast search using the parasite specific MEME motifs confirmed the specificity to the parasite proteins (see Additional file 2: Table S1).

All these results highlighted higher similarities between the two parasite proteins and the *hu*MTAP as compared to *hu*PNP, with a higher relatedness between *Li*MTAP and *Tb*MTAP than with the human proteins. This was strengthened by the presence of specific motifs on the parasite MTAP sequences and specificities within the active site of the *hu*MTAP that may be further exploited.

### 3D structure modeling revealed parasite MTAP vs. *hu*MTAP differences

A thorough structural analysis was generated for *Li*MTAP and *Tb*MTAP through the I-TASSER server [44]. Interestingly, templates used for building the parasite protein 3D homology models were the same and corresponded to the putative bacterial MTAP structures having PDB IDs: 4GLF and 4GLJ [25]. Five models were proposed for each protein. For both MTAPs, C-score of the first model among the five generated was significantly higher than those of the remaining models (see Additional file 3: Table S2). Thus, model 1 was considered as the best one in both cases, and was retained for this study. The 3D model of *Li*MTAP had a C-score of 1.17, an estimated TM-score of 0.87 ± 0.07 and an estimated root-mean-square deviation RMSD of 3.8 ± 2.6 Ǻ. The 3D model of *Tb*MTAP had a C-score of 0.96, an estimated TM-score of 0.84 ± 0.08 and an estimated RMSD of 4.3 ± 2.9 Ǻ.

The parasite protein models were used as query structures to parse the PDB database for structurally related entries. Resulting protein structures were the same for *Li*MTAP and *Tb*MTAP models, and corresponded either to MTAPs or to PNPs (Table 1). Their TM-scores exceeded 0.5, reflecting a structural closeness to the target proteins (*Li*MTAP and *Tb*MTAP) as the TM-score is a length-independent metric that measures the global fold similarity between two proteins, with low sensitivity to local structural variations [51]. Moreover, RMSD of atomic positions were somewhat lower for MTAP entries compared to PNP entries (Table 1). Thus, *Li*MTAP and *Tb*MTAP proteins presented higher similarity to MTAPs than PNPs.

**Table 1:** Data related to the ten structural analogs of *Li* (a) and *Tb* (b) MTAP 3D models identified by I-TASSER

PDB ID	Protein	Ligand	Protein stoichiometry	Organism	TM-score (a)	RMSD (a)	TM-score (b)	RMSD (b)
4GLF	MTAP	–	Homo trimer	Cultured bacterium	0.929	0.53	0.919	0.56
1WTA	MTAP	ADE + PO_4_	Homo trimer	*Aeropyrum pernix*	0.830	1.84	0.826	1.84
1V4N	MTAP	–	Homo trimer	*Sulfolobus tokodai*	0.828	1.82	0.822	1.77
3 T94	MTAP	MTA + SO_4_	Homo hexamer	*Sulfolobus solfataricus*	0.821	1.87	0.814	1.86
3OZC	MTAP	4CT + PO_4_	Homo trimer	*Homo sapiens*	0.811	1.91	0.805	1.89
4L5A	MTAP	TBN + SO_4_	Homo trimer	*Schistosoma mansoni*	0.805	2.19	0.798	2.07
3OZB	MTAP	HPA + SO_4_	Homo hexamer	*Pseudomonas aeruginosa*	0.744	1.73	0.738	1.71
1TCV	PNP	NDS + ACT	Homo trimer	*Schistosoma mansoni*	0.715	2.97	0.712	2.99
2P4S	PNP	DIH + PO_4_	Homo trimer	*Anopheles gambiae*	0.711	2.98	0.707	3.02
1A9O	PNP	PO_4_	Homo trimer	*Bos taurus*	0.709	3.05	0.707	3.22

I-TASSER server also provided EC number [52] predictions for *Li*MTAP and *Tb*MTAP. It returned five homologous enzymes for each target. In both cases, four enzymes had 2.4.2.28 as EC number, which correspond to MTAPs and one enzyme, with the lowest TM-score, having 2.4.2.1 as EC number, which corresponds to a PNP (see Additional file 4: Table S3). This brings additional confirmation on the annotation of these proteins as 5'-methylthioadenosine phosphorylases.

**Fig. 3:**
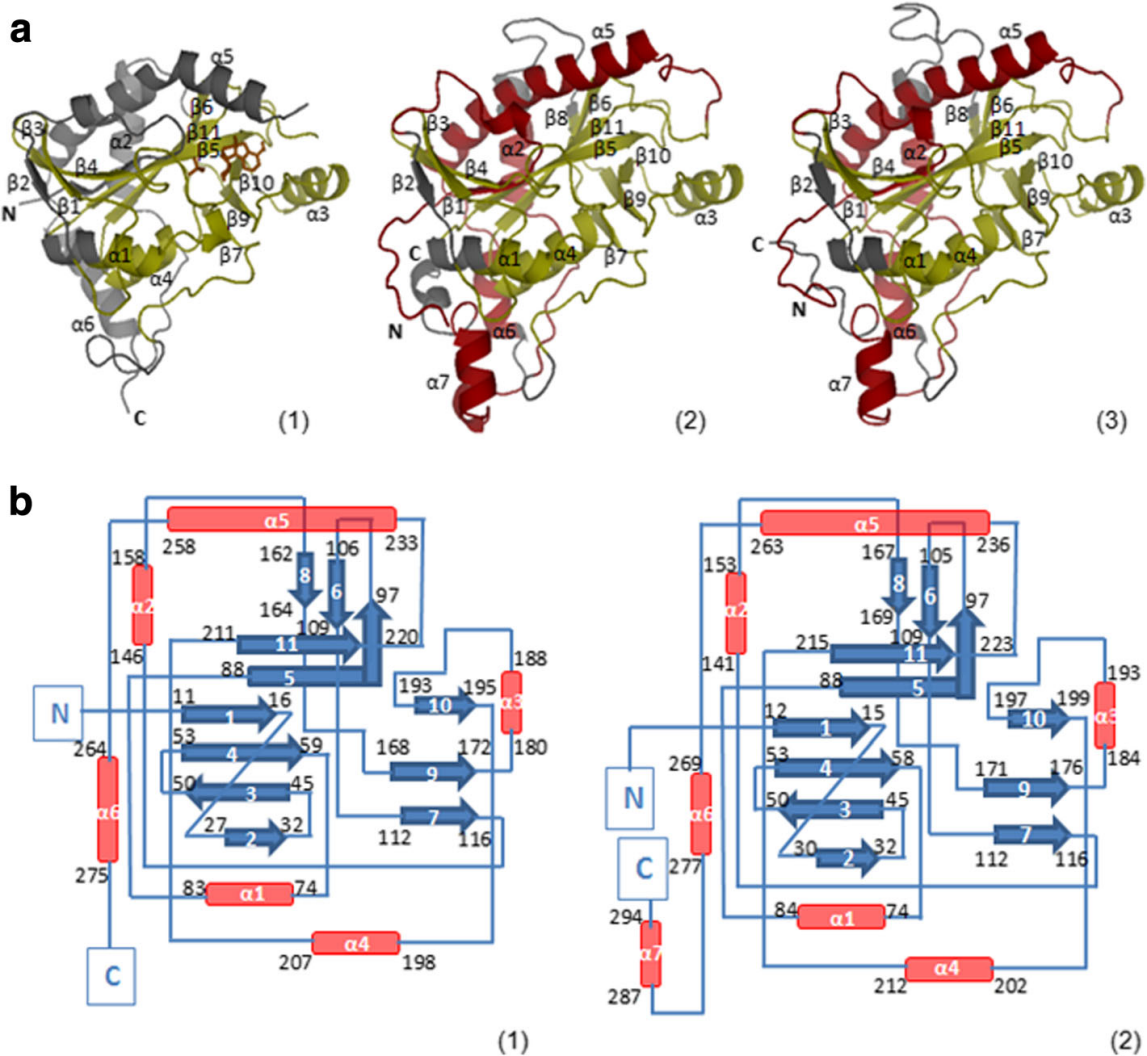
Three- dimensional models and topology of (1) *hu*MTAP (pdb: 1CG6), (2) *Li*MTAP and (3) *Tb*MTAP. **a** The cartoon representation of the 3D models integrates the MEME motifs, which are represented in different colors: yellow for the conserved MEME motifs and red for the parasite specific ones. The protein parts that do not include a MEME motif are colored in grey. The secondary structure elements are labeled on each MTAP model. The N- and C- terminal extremities are marked by N and C, respectively. **b** Only the topology diagrams of (1) *hu*MTAP and (2) *Li*MTAP are displayed as the *Tb*MTAP diagram is identical to the *Li*MTAP one. On these diagrams, the α-helices and β-strands are shown by red cylinders and blue arrows, respectively, and are labeled by numbers starting from the N- terminal extremity. The first and last amino acids of each secondary structure are shown on the corresponding shape, on both *hu*MTAP (1) and *Li*MTAP (2) diagrams. The connector coils are shown as blue lines. On the parasite protein topology diagrams, the α7 helix brings the C- terminal extremity in a spatially close position to the N- terminus

The *Li*MTAP and *Tb*MTAP models were further refined using the ModRefiner server. Refined models returned lower RMSD values and higher TM-scores in comparison to initial models (see Additional file 5: Table S4). We performed this step in order to verify that the models that will be used for the next steps present energetically minimized structures. We aligned the refined parasite MTAP structure models on chain A of the PDB entry 1CG6, which corresponds to the *hu*MTAP protein co-crystallized with its natural substrate (MTA) and a sulfate ion (SO_4_^2−^) as a co-factor [18]. The three proteins aligned perfectly with high conservation of the secondary structures (see Additional file 6: Figure S2), except for the coil at the N-terminus, the coil linking the α2 helix to the β8 strand and the α7 helix located on the C-terminus of the parasite MTAPs (Fig. 3a). These structural differences also matched high divergence regions on the PSA, i.e., the N- and C- termini and the central region covering residues from 150 to 166 in *Li*MTAP sequence. This added confidence to our primary sequence alignment at these divergent regions, which were also part of the parasite specific M4, M5, M7 and M8 motifs (Figs. 1 and 2).

The models of *Li*MTAP and *Tb*MTAP also presented high secondary structure conservation with the *hu*MTAP (Fig. 3b). All three structures of the MTAP proteins presented the same number of β sheets (11), whereas the parasite proteins presented one extra α helix (α7) as compared to the human counterpart, which only counts six α helices (Fig. 3b). Each secondary structure element on the sequences of the parasite proteins matched its counterpart on the human protein sequence, as shown in Fig. 1. Noticeably, the α7 helix appeared at the C-terminal region of the parasite proteins, the most divergent region on the PSA, which corresponds to a C terminal extension on these proteins. On the topology diagrams, this helix brings the C-terminal region of the parasite proteins in close proximity to the N-terminus (Fig. 3b). This is noteworthy, as it may induce significant differences as compared to the *hu*MTAP, provinding a basis for potential specific molecular interactions within a monomer or in the multimeric protein. Moreover, the α7 helix overlapped with MEME M8 motif, identified as specific to *Li*MTAP and *Tb*MTAP (Fig. 2a). In the same line, the motifs identified as specific to the parasite proteins (M4, M5, M7 and M8) mostly covered α helices (Table 2) that are exposed on the protein surfaces (Fig. 3a). More importantly, the conserved motifs (M1, M2, M3 and M6) mostly covered β sheets (Table 2), described in the literature as implicated in the active site and/or in trimeric contacts within the subunits of the NP-I family members [15]. On both models of *Li*MTAP and *Tb*MTAP, these β sheets formed a central ensemble surrounded by α helices as shown in Fig. 3b. This indicated that both proteins belong to the α/β structural class and to Rossmann fold topology according to the PDB annotations (http://www.rcsb.org). This fold is common to all NP-I family members [15], including the *hu*MTAP (Fig. 3a). All these results demonstrated that our modeled parasite proteins are members of the NP-I family with more closeness to MTAPs than to PNPs. Structural peculiarities correlated with MEME modeling of parasite specific motifs and divergent primary sequence are also highlighted.

**Table 2:** Functional or structural correlation of MEME sequence motifs found in *hu*MTAP, *Li*MTAP, *Tb*MTAP and *hu*PNP

Motif	Involved secondary structures	Structural/functional significance in NP-I family ^(a)^
Common MTAP motifs		
M1	α3, β10, α4, β11	Involved in active site and trimeric contacts
M2	β3, β4, part of α1	Involved in active site
M3	β5, β6, β7	Involved in active site and trimeric contacts
M6	β8, β9	
Parasite specific motifs		
M5	β1	Involved in active site
M7	part of α2	
M4	α5, α6	
M8	α7	

^(a)^Reviewed by Pugmire and Ealick [15]

### Identification of differently sized active sites

**Fig. 4:**
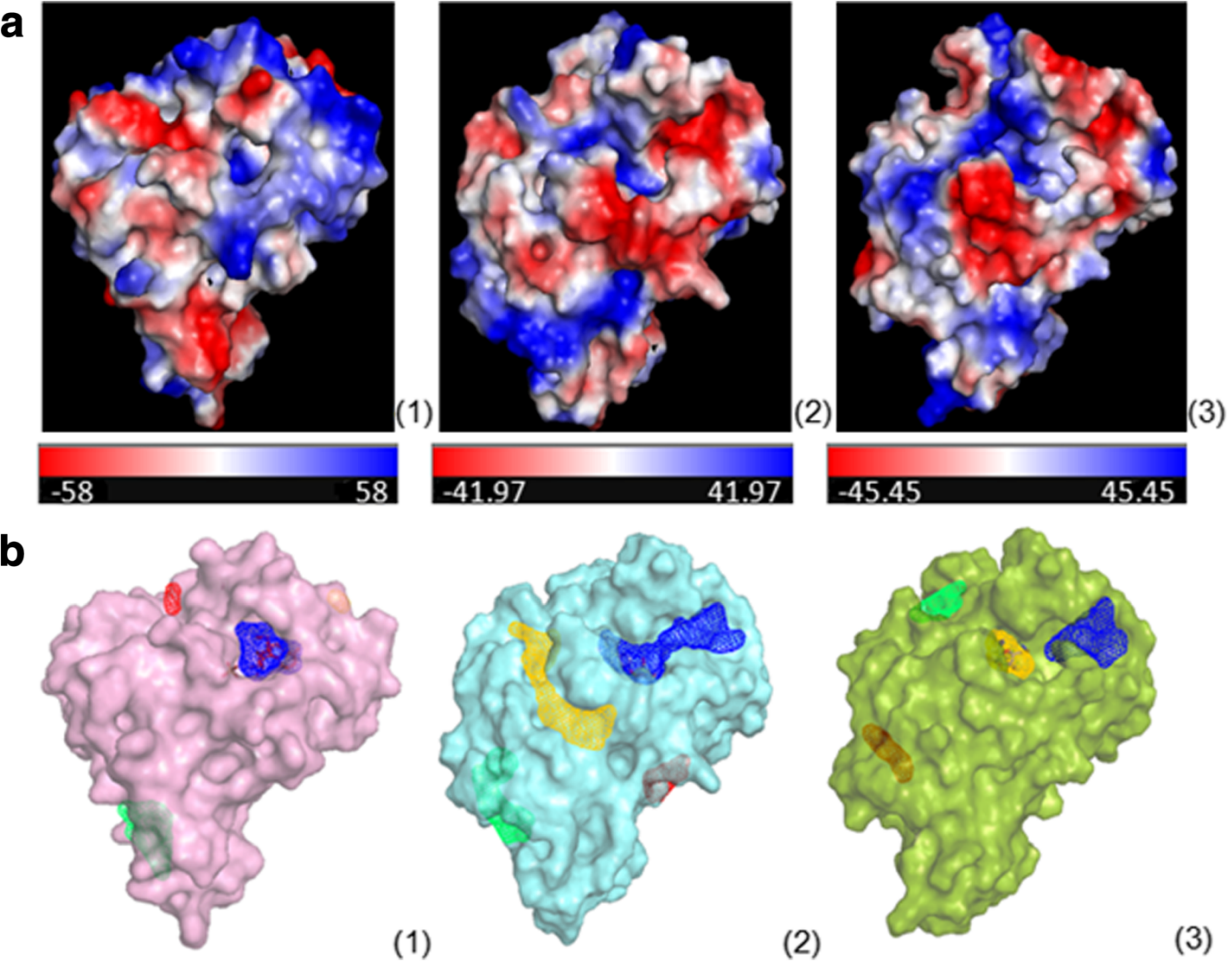
Solvent-accessible surface area (SASA) of the three MTAP proteins and potential binding sites. **a** The Electrostatic Potential (EP) projected on proteins SASA is presented in red-white-blue color gradient for negatively, neutral and positively charged regions, respectively. The range of its variation is also presented. **b** Four binding sites (BSs) were identified by SOM-BSfinder on the SASA of the three MTAPs, each having a different surface color (pink, cyan, or green). They were ranked and colored according to descending cluster size from blue (highest neuron density associated with low U-values) to green, yellow and red (lowest neuron density associated with high U-values). The panels represent: (1) *Hu*MTAP, (2) *Li*MTAP and (3) *Tb*MTAP

The three MTAP proteins (*Li*MTAP, *Tb*MTAP and *hu*MTAP) were further compared by analysis of their surfaces and electrostatic properties. Solvent accessible surface areas (SASA) were generated for the three MTAPs and the adaptive Poisson-Boltzmann solver (APBS) was used to calculate their electrostatic potentials (EPs) (Fig. 4a). EP distributions on *Li*MTAP and *Tb*MTAP surfaces presented equivalent ranges, varying from −42 to +42 and from −45 to +45, respectively (Fig. 4a). However for *hu*MTAP, it presented a wider range varying from −58 to +58 (Fig. 4a). As the EP depends on the nature of the protein residues, these differences may be explained by the fact that the parasite proteins presented more identity to each other than to their human counterpart. However, globally the charge distribution on these proteins was different for each one and unique to it.

The protein surfaces also contained different cavities and pockets (Fig. 4b). In order to define and characterize the ASs and other potential BSs on the parasite protein surfaces, we used the SOM-BSfinder algorithm [45]. This probe-mapping method identifies potential BSs as “high neuron consensus”- areas on a 3D map. Four BSs were identified for each target (Fig. 4b). For *hu*MTAP, the first site identified by SOM-BSfinder, BS1, (shown in blue on Fig. 4b) matched the AS defined by the location of the MTA on the co-crystal structure (PDBid: 1CG6) [18]. The first site (BS1) identified on *Li*MTAP (shown in blue on Fig. 4b) occurred in a cavity having the same location as the AS of the *hu*MTAP, when both proteins were aligned (Fig. 4b, see Additional file 6: Figure S2). Thus, BS1 was considered as the AS of the *Li*MTAP. In the case of *Tb*MTAP, both BS1 (blue) and BS3 (yellow) (Fig. 4b) occurred at the cavity that corresponded to the AS of *hu*MTAP (Fig. 4b). This suggested that both BSs jointly constituted the AS of *Tb*MTAP, as they occupied two parts of the same cavity. Both proteins, *Li*MTAP and *Tb*MTAP, presented larger AS cavities than the *hu*MTAP one, which may confer to them specific geometric and physical properties.

These results further point to predicted peculiarities affecting the surface of the proteins and particularly the active sites as a result of global sequence divergence and surface charge distributions.

### Molecular docking revealed different ligand binding modes and atomic interactions

Given the differences depicted between the three MTAP proteins at the structural level in general and at the AS in particular, we performed molecular docking of MTA on their ASs in order to predict potential impact on the binding modes and interactions with ligands.

**Fig. 5:**
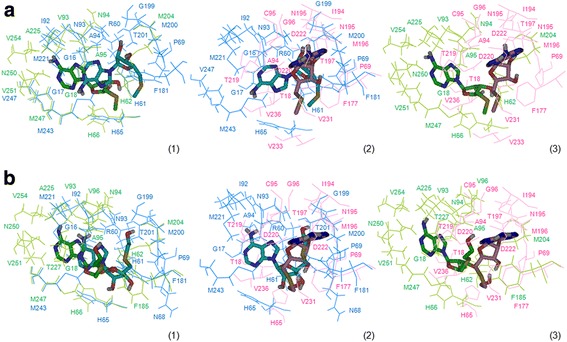
Residues involved in MTA and HETA binding into the AS of the three MTAPs. MTA and HETA were docked into the ASs of the 3 proteins and the best docking poses of each ligand on each protein are shown along with residues within 4Ǻ distance. The Figures were generated when all three proteins were aligned, and from the same view angle. Panel (**a**) shows residues involved in MTA binding. Panel (**b**) shows residues involved in HETA binding. The panels show superposition of residues in presence of the docked ligand: (1) from *Li*MTAP (cyan) and *Tb*MTAP (green); (2) from *Li*MTAP (cyan) and *hu*MTAP (magenta); (3) from *Tb*MTAP (green) and *hu*MTAP (magenta)

Docking of MTA on the *hu*MTAP was performed on the corresponding crystal structure (PDB entry: 1CG6). The lowest-energy pose was very similar to the crystal structure with a good docking score of −7.8. AutoDock vina has been identified by the Community Structure-Activity Resource (CSAR) [53] as one of the most interesting tools in identifying the accurate binding mode of a molecule and predicting the corresponding binding affinity. Thus, we considered the lowest-energy docking poses of MTA on the parasite proteins as the most relevant ones. Docking scores of MTA were of −6.1 and −5.8 on *Li*MTAP and *Tb*MTAP, respectively. These poses correspond to equivalent binding modes of MTA on *Li*MTAP and *Tb*MTAP, which differ from the binding mode on *hu*MTAP (Fig. 5a). The purine ring of MTA was oriented to the left on the ASs of the parasite proteins and to the right on the *hu*MTAP (Fig. 5a). In both cases, this part of the MTA molecule was interacting with the negatively charged areas on the corresponding SASA (Fig. 4a).

Residues involved with MTA binding onto the ASs of the three proteins, denoted herein as interacting residues (IRs), were defined as those presenting at least one atom potentially involved in an interaction with at least one ligand atom, at a distance lower than 4 Ǻ. This cutoff was set up according to choices used by other groups to define a potential interaction [18, 34]. The nature of the interacting atoms was examined in order to validate the interaction type. We identified structurally equivalent IRs (interacting residues that spatially superimposed) when the protein 3D structure/models were aligned (Fig. 5a). On *Li*MTAP, ten IRs, G17, H61, H65, I92, N93, A94, M200, M221, M243 and V247, had their structural equivalent on *Tb*MTAP: G18, H62, H66, V93, N94, A95, M204, A225, M247 and V251, respectively (Fig. 5a, Table 3). Six residues on *Li*MTAP, P69, A94, F181, G199, M200 and T201 had their structural equivalent on *hu*MTAP: P69, A94, F177, N195, M196 and T197, respectively (Fig. 5a). Noticeably, only the two residues A94 and M200 on *Li*MTAP structurally aligned simultaneously on *hu*MTAP and *Tb*MTAP (Table 3). Obviously, the *hu*MTAP showed ten specific residues that had no structurally equivalent IRs on the parasite proteins: T18, C95, G96, I194, T219, D220, D222, V231, V233 and V236 (Table 3, Fig. 5a). The T18 residue was described in the literature as interacting with the co-factors SO_4_/PO_4_ [15, 18], while it appeared herein as interacting with the sulfur atom of MTA at a distance ~ 3.85 Ǻ. On the crystals, residues T219, D220 and D222 interact with the purine ring of MTA, and V233 and V236 are involved in hydrophobic interactions with the methylthio-ribose [15, 18]. These MTA interactions were confirmed through our docking results. Residues C95, G96, I194 and V231 were not described in the literature but appeared through our protocol as IRs. In our docking results and on the crystal pose of MTA, these four residues, along with T18, appeared at a distance higher than 3.8Ǻ, which may explain why they were not considered as relevant interactions by the crystallographers [15, 18]. Herein, we considered all possible interactions as relevant in order to minimize false negatives.

**Table 3:** Residues involved with MTA binding to the three MTAPs

*hu*MTAP	–	–	T18^a^	–	–	**–**	P69^b^	–	–	A94^a^	C95^b^	G96^b^	F177	I194^b^	N195^b^	M196	T197^a^	T219	D220	D222	–	–	V231^b^	V233	V236	–	–	–
*Li* MTAP	G16	G17	–	R60	H61	H65	P69	I92	N93	A94	–	–	F181	–	G199	M200	T201	–	–	–	M221	M243	–	–	–	–	V247	–
*Tb* MTAP	–	G18	–	–	H62	H66	–	V93	N94	A95	**–**	–	**–**	–	–	M204	–	–	–	–	A225	M247	–	–	–	N250	V251	V254
MEME motifs	5	5	–	2	2	2	2	3	3	3	3	3	1	1	1	1	1	1	1	1	1	4	–	–	–	4	4	4

The table illustrates interacting residues (IRs) with MTA at a distance lower than or equal to a cutoff of 4Ǻ. Residues listed in the same column are structurally aligned IRs. As we did not dock the cofactor on the proteins, it was expected not to observe interactions on *hu*MTAP involving the cofactor binding sites (R60, H61, T93). However, three cofactor- binding sites (^a^) were here identified as IR with MTA. (^b^) Corresponds to residues here identified as IRs but not on the crystal structure of *hu*MTAP

On our model, the residues of *hu*MTAP interacting with the purine ring of MTA were F177, T219, D220 and D222, which is consistent with literature [18]. Except for F177, these residues had no structurally equivalent IRs on the parasite proteins (Table 3) although they matched perfectly well on the primary sequence alignment. The residues interacting with the purine ring of MTA were G16, G17, R60, H65, I92, N93, A94, M221, M243 and V247, in *Li*MTAP and G18, V93, N94, A95, A225, M247, N250, V251 and V254, in *Tb*MTAP (Fig. 5a). Of these residues, I92, M221, M243 and V247 on *Li*MTAP and their counterparts (V93, A225, M247 and V251) on *Tb*MTAP appeared to establish hydrophobic interactions with the two rings of the purine moiety of MTA (Fig. 5a). This type of hydrophobic pocket/interactions have been depicted with other NP-I family members, namely *E. coli* Uridine Phosphorylase [54, 55]. Interestingly, the residue M196 in *hu*MTAP was interacting with the ribose ring of MTA [18]. Its structural counterparts in *Li*MTAP (M200) similarly established a hydrogen bond with the 2′- hydroxyl of the ribose (Fig. 5a). However, their trypanosomal counterpart (M204) was involved in hydrophobic interactions with the 5′-methylthioribose part of MTA (Fig. 5a).

In a second step, we docked HETA, a specific inhibitor of *Tb*MTAP [10, 35], on all three proteins (Fig. 5b). The lowest-energy docking poses of HETA had scores of −7.5, −6.2 and −5.9 respectively on *hu*MTAP, *Li*MTAP and *Tb*MTAP. These poses presented similar binding modes to the ones obtained for MTA, with opposite purine orientations of docked HETA on the parasite proteins as compared to *hu*MTAP (Fig. 5b). For *Li*MTAP, only residue N68 appeared as specifically interacting with HETA vs. MTA, and V247 as specifically interacting with MTA vs. HETA (Tables 3 and 4). For *hu*MTAP, only residue H65 appeared as specifically interacting with HETA vs. MTA and residues R60, H61, T93 and V233 appeared as specific to the interaction with MTA vs. HETA. For *Tb*MTAP, three residues V96, F185 and T227 appeared as specific to HETA vs. MTA (Tables 3 and 4). They interacted with the ethylthio-ribose part of HETA, which includes an additional ethyl group compared to MTA (see Additional file 1: Figure S1), (Fig. 5b). No such specific interactions could be predicted for *Li*MTAP and *hu*MTAP (Tables 3 and 4, Fig. 5b). This is consistent with HETA being a specific inhibitor to *T. brucei* [10, 35].

**Table 4:** Residues involved with HETA binding to the three MTAPs

*hu*MTAP	–	–	T18	–	–	**H65**	–	P69	–	–	A94	C95	G96	F177	I194	N195	M196	T197	T219	D220	D222	–	–	–	V231	V236	–	–	–
*Li* MTAP	G16	G17	–	R60	H61	H65	**N68**	P69	I92	N93	A94	–	–	F181	–	G199	M200	T201	–	–	–	M221	–	M243	–	–	–	–	–
*Tb* MTAP	–	G18	–	–	H62	H66	–	–	V93	N94	A95	**V96**	–	**F185**	–	–	M204	–	–	–	–	A225	**T227**	M247	–	–	N250	V251	V254
MEME motifs	5	5	–	2	2	2	2	2	3	3	3	3	3	1	1	1	1	1	1	1	1	1	1	4	–	–	4	4	4

The table illustrates interacting residues with HETA at a distance lower than or equal to a cutoff of 4Ǻ. Residues listed in the same column are structurally aligned IRs. Residues were observed that interact in the three MTAPs with both MTA and HETA. Residues specifically involved with HETA binding (vs. MTA) are shown in bold

In spite of the active site residues conservation with *hu*MTAP, our results indicated binding modes that are specific to the parasites proteins due to the striking differences predicted on the surface of these proteins. Moreover, qualitative variations in molecular interactions -with different ligands- are also pointed to within the AS itself as a result of the global sequence divergence. This highlights relevance of natural diversity of *Li*MTAP and *Tb*MTAP in shaping structural and functional differences.

### A peptide unique to *Li*MTAP could be specifically targeted by a polyclonal antibody

**Fig. 6:**
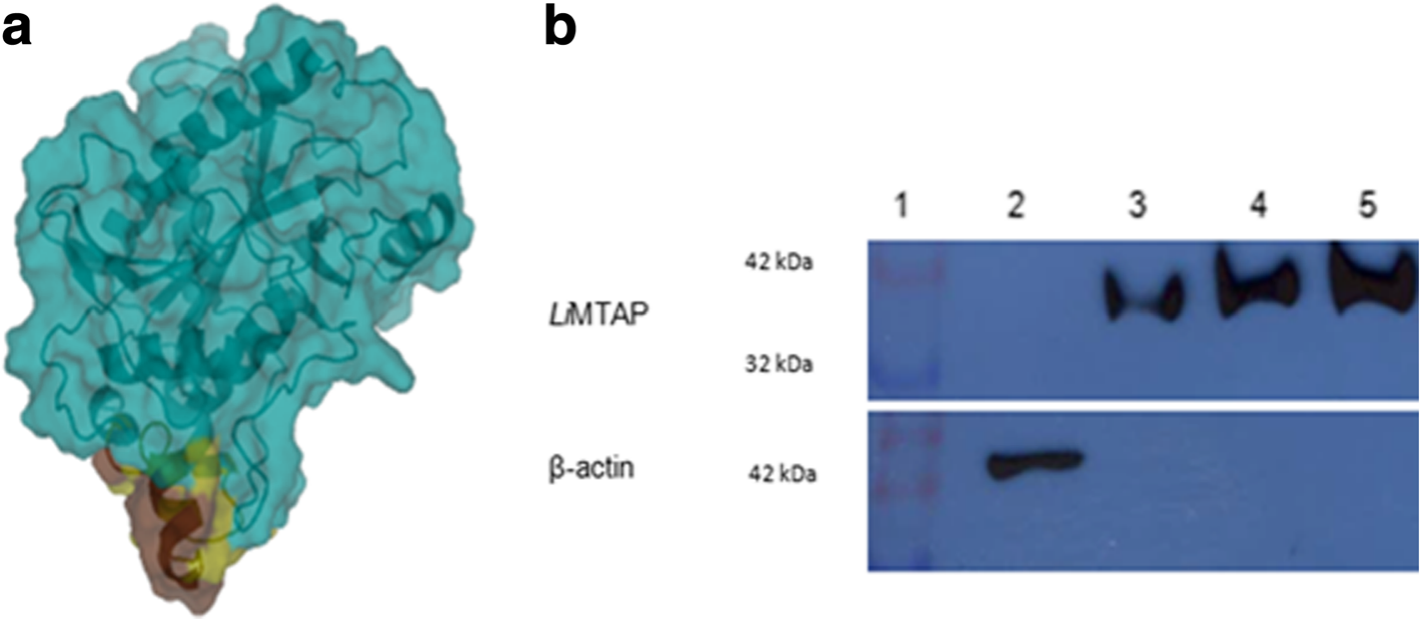
Characterization of the polyclonal antibody directed against an antigenic C-terminal peptide of *Li*MTAP. **a** C-terminal location of the surface antigenic peptide unique to *Li*MTAP. The peptide includes amino acids 277 to 300, colored in yellow or brown. Residues identified as highly exposed through I-TASSER (E283, H284, A287, E288, Q291, S299) are colored in brown. **b** Specificity of the C-terminal *Li*-peptide was tested by western blot using respectively the *Li*MTAP antibody, and the β-actin antibody as a control for human PBMC lysates. The total proteins extracted from *Leishmania* and human PBMC were resolved on 12% SDS-PAGE gel, transferred to PVDF membrane and then subjected to western blot analysis using anti-*Li*MTAP (1/10000) or anti-β-actin (1/5000) antibodies. The Figure is representative of three independent experiments. Lanes: (1) Prestained marker MW in kDa (Vivantis, CA, USA); (2) Human PBMC lysates; (3), (4) and (5): 3, 7 and 15 micrograms of *L. infantum* (LV50) promastigote lysates, respectively

Based on differences observed at the primary sequence and the 3D structure levels between *Li*MTAP and *hu*MTAP, we selected four peptides on *Li*MTAP presenting high divergence between both proteins of which 3 corresponded to specific MEME (M4, M5, M8) motifs (Table 5). Peptide number 4 (also corresponding to motif M8) was chosen, according to Genscript recommendations, for having the highest solvent accessibility and antigenicity, to generate an antibody that should be specific to *Li*MTAP. Peptide 4 included amino acids from 277 to 300 (AIVTKPEHIPAETKQRIAPLVASK), covered the α7 helix on the C-terminus of *Li*MTAP and comprised six residues identified by I-TASSER as highly exposed, namely E283, H284, A287, E288, Q291 and S299 (Fig. 6a). An antibody was successfully generated against this peptide.

**Table 5:** List of the four peptides selected *in silico* on *Li*MTAP and their correlation to MEME motifs

Peptides	Amino acids sequence (position)	MEME motifs
1	MYGNPHKEPVAIAV (1–14)	M5
2	HEALLRCFPDVAAGKGTFQIH (148–168)	–
3	DAPHVDAAQVTKV (230–242)	M4
4	AIVTKPEHIPAETKQRIAPLVASK (277–300)	M8

The total *Leishmania* proteins were extracted, separated on 12% SDS PAGE and transferred to polyvinylidene difluoride membrane and then subjected to western blot analysis using the anti-*Li*MTAP directed against peptide 4. As a control, we carried out the same experiment on human PBMC lysates. The results showed that anti-*Li*MTAP antibody recognized only the *Leishmania* protein extract. No signal was detected with the human PBMC extracts, whereas, they were recognized by the anti-β actin antibody (Fig. 6b). Similar observations were also made using lysates of the THP1 cell line (see Additional file 7: Figure S3).

These results showed that the anti-*Li*MTAP antibody was specific to the *Li*MTAP protein and suggested the possibility to exploit the structural differences for the development of specific anti-*Leishmania* biomolecules.

## Discussion

Leishmaniases are neglected tropical diseases having a worldwide distribution and till to date there are no effective vaccines available to prevent them [2]. Mainstay and second line drugs currently used for the treatment of leishmaniasis have serious side effects; resistance to antimony or miltefosine is also increasingly reported [5, 56]. Therefore, search for alternative drugs to treat leishmaniasis is a research priority. The metabolic pathways of *Leishmania*, which are either absent or different from the mammalian host and involved in survival, pathogenesis or drug resistance of the parasite, constitute excellent potential targets for the rational design of antiparasitic drugs [8, 57]. Several reports have shown that targeting the polyamine biosynthesis and purine salvage enzymes against *Trypanosomatidae* have yielded promising results [10, 14, 35, 58]. Among these enzymes, MTAP plays a crucial role in purine and polyamine metabolism and in the methionine salvage pathway [10]. Selective inhibitors of the *T. brucei* enzyme have been described, and they showed an in vitro cytotoxicity (IC_50_) of 10 nM for the 5′-deoxy-5′-(hydroxyethylthio)-tubercidin and high cure rates (70% to 90%) of HETA when administrated to mice infected with *T. brucei* [10, 35]. However, to our knowledge, in *Leishmania*, an organism phylogenetically close to *Trypanosoma*, the protein MTAP was not considered so far as a potential drug target and was not characterized yet. Given the role of this protein in humans and its putative functional conservation in the studied parasites, it seemed important to characterize the protein in *Leishmania infantum*, a pathogen that causes visceral leishmaniasis in North Africa, Europe, Asian countries and Latin America. Indeed the knowledge of the 3D structure of a drug target protein is of great importance to conduct structure-based drug discovery. Such characterization allows confirming putative roles and identification of commonalities as well as specific features to an organism, and thus assessing whether natural diversity of such conserved proteins could make them potentially good candidates for drug design. When a protein structure is not or could not be resolved experimentally, homology modeling is one of the most powerful tools to obtain robust models of a protein structure [59, 60].

Bioinformatics approaches were used to bring insights into the trypanosomal sirtuin structure and function from *L. major, L. infantum, T. brucei* and *T. cruzi*. Structure comparisons with the human protein and molecular docking permitted to highlight specificities that were of interest in predicting specific/selective inhibitors [61]. The *Leishmania* elongation factor alpha, sharing 82% of identity with its mammalian orthologue, was also successfully used in identifying novel anti-*Leishmania* molecules *in silico* [62]. In spite of this high identity rate, selective inhibitors could be identified *in silico* that targeted a unique structural feature on the parasite protein, resulting from a 12 amino acids long deletion [62]. Herein, we used homology modeling to generate the 3D structure models of *Li*MTAP and *Tb*MTAP and compare them to other MTAP structures of relevance like in humans. For this purpose, 3D models of the parasite MTAPs were performed by I-TASSER, matching structure predictions with known functional templates [41]. This homology modeling server was already used by other groups for instance to understand functions of human thiol dioxygenase enzymes [63] and to assess stability of the Rabies Virus G protein trimer through molecular dynamics [64].

Through sequence and structure comparison of the putative *L. infantum* MTAP protein with the human and *T. brucei* counterparts, we could depict significant global similarities. Important sequence identity rates, level of active site residues conservation, presence of common MEME motifs to NP-I family members, similar 3D topology, and I-TASSER functional annotations and EC number predictions (2.4.2.28) consolidated the hypothesis that the *Leishmania* protein is a 5′-methylthioadenosine phosphorylase. However, in spite of these commonalities shared by the three proteins, it was possible to identify specific structural features that were congruent with divergence on the primary sequence, itself underlying specific MEME modeled motifs. Relevance of such structural specificities was further confirmed by the identification of a highly antigenic and exposed peptide among four structurally divergent regions/peptides corresponding to one of these specific motifs. Notably, a polyclonal antibody directed against this peptide at the C-terminus proved to recognize specifically the *Leishmania* protein (and so did not react with human cell extracts).

Primary sequence divergence, reflected on surface EP and BS predictions, brought additional information about peculiarities of the parasite protein models. Notably, the predicted active sites (located on similar parts of the proteins) presented different shapes and volumes that prompted looking at the protein-ligand interactions. Indeed, these are of high importance towards a comprehensive study of enzymes in general and drug targets in particular [24, 61, 65]. Molecular docking of MTA, the natural substrate, and HETA, a well characterized inhibitor in *T. brucei*, into the AS of the three proteins showed equivalent docking scores between MTA and HETA and lower docking scores (better free energy of binding) on the human target as compared to the parasitic counterparts. This could be due to the fact that docking simulations were performed on a crystal structure of the *hu*MTAP, which corresponds to the biologically optimal conformation for ligand binding. Molecular docking of both MTA and HETA highlighted differential binding modes where the purine ring occupied opposite position in parasite MTAPs to the one in the human protein. The interactions into these pockets defined within a 4 Ǻ range, corresponding to hydrogen bonds or hydrophobic interactions, also revealed qualitative differences at different levels in each comparison. Notably, the docking of MTA molecule predicted specific IRs in *hu*MTAP that had no structural equivalent IRs on the parasite MTAPs. Moreover, the docked MTA into both *Li*MTAP and *Tb*MTAP involved less hydrophobic IRs with the methylthio part and much more interactions with the purine than those seen in *hu*MTAP. In addition, the interactions with the ethyl group of HETA were characterized by three residues (V96, F185 and T227), only seen in *Tb*MTAP and not in its *Leishmania* and human counterparts. Noticeably, most residues that are unique to *Leishmania* or *Trypanosoma* protein belong to kinetoplastid specific MEME motifs. These were essentially mapping on the surface of the protein and embedding α helices, while the ones shared with the *hu*MTAP were mostly within the central β core. Importantly, our analysis also brought structural explanation to the specific inhibitory effect of *Tb*MTAP by HETA [10], through the presence of specific HETA IRs within the *Tb*MTAP AS. This approach could constitute a basis for the design of non-active mutants and/or the design of transition-state inhibitors [24, 35]. In line with this, a recent structural study of the MTAP of *Schistosoma mansoni* (*Sm*) highlighted structural features that differentiated this protein from human MTAP, bringing basis for intelligent design of novel *Sm*MTAP inhibitors [24].

## Conclusions

In conclusion, our study highlights commonalities and peculiarities among human, *L. infantum* and *T. brucei* MTAP proteins. Primary, secondary and tertiary alignments correlated well to each other in spite of local sequence divergence. Herein, we put an emphasis on such divergence as it has a functional relevance among naturally occurring MTAPs. The study predicts structural differences that may impact enzymatic activities of the *Leishmania* protein in presence of the natural substrate or other ligands. It also refers that sequence peculiarities could be targeted to design *Leishmania* specific biomolecules. This is a first step towards selection of *Leishmania* MTAP as a potential drug target.

## Additional files

Additional file 1: Figure S1. Chemical structure of MTA and HETA, docked in *Hu*MTAP, *Li*MTAP and *Tb*MTAP active sites. (PPTX 66 kb)

Additional file 2: Table S1. Taxonomy blast reports for MEME motifs. Organism, blast name, score, number of hits and organism description were provided for each MEME motif report. (a) M5 motif, (b) M7 motif, (c) M4 motif, (d) M8 motif, (e) M6 motif. (PPTX 974 kb)

Additional file 3: Table S2. C-scores of the five models generated by I-TASSER for *Li*MTAP and *Tb*MTAP. The first model was retained for each protein (*Li*MTAP and *Tb*MTAP) as it presented the highest C-score. (PPTX 44 kb)

Additional file 4: Table S3. EC number predictions provided by I-TASSER for *Li*MTAP and *Tb*MTAP 3D models. Four hits among the five returned had the highest TM-scores and 2.4.2.28 as EC number, which corresponds to 5’-Methylthioadenosine phosphorylase. (PPTX 86 kb)

Additional file 5: Table S4. RMSD (Ǻ) and TM-score of the refined 3D models of *Li*MTAP and *Tb*MTAP. Refined models returned low RMSD values and high TM-scores. (PPTX 39 kb)

Additional file 6: Figure S2. Alignment of *Li*MTAP and *Tb*MTAP 3D models on the Human crystal structure (PDB: 1CG6). *Hu*MTAP*, Li*MTAP and *Tb*MTAP were represented by cartoons and colored in violet, cyan and yellow, respectively. The three MTAP models aligned perfectly. (PPTX 151 kb)

Additional file 7: Figure S3. Characterization of the polyclonal antibody directed against an antigenic C-terminal peptide of *Li*MTAP. The total proteins extracted from *L. infantum* and human THP1 cells were resolved on 12% SDS-PAGE gel, transferred to PVDF membrane and then subjected to western blot analysis using anti-*Li*MTAP (1/10000) antibody. The Figure is representative of three independent experiments. Lanes: (1) Prestained marker MW in kDa (Vivantis, CA, USA); (2) THP1 lysates; (3) Fifteen micrograms of *L. infantum* (LV50) promastigote lysates. (PPTX 45 kb)

## Abbreviations

3D: Three dimensional
APBS: Adaptive Poisson-Boltzmann Solver
AS: Active site
BS: Binding site
CL: Cutaneous leishmaniasis
EC: Enzyme commission
EP: Electrostatic potential
GO: Gene ontology
HETA: 5′-hydroxyethylthio-adenosine
Hu: Human
IR: Interacting residues
*L. major*: *Leishmania major*
*Li*: *Leishmania infantum*
MTA: 5′-methylthioadenosine
MTAP: 5′-methylthioadenosine phosphorylase
MTM: Methylthio-immucillin-A
NP-I: Nucleoside phosphorylase I
NP-II: Nucleoside phosphorylase II
NTD: Neglected tropical disease
PBMC: Peripheral blood mononuclear cells
PNP: Purine nucleoside phosphorylase
PSA: primary sequence alignment
SASA: Solvent accessible surface area
SOM: Self-Organized Map
*Tb*: *Trypanosoma brucei*
TDR: Tropical Disease Research
VL: Visceral leishmaniasis
