# Supplementary figures and images for: Leishmania infantum 5’-Methylthioadenosine Phosphorylase presents relevant structural divergence to constitute a potential drug target
Source: BMC Struct Biol. 2017 Dec 19;17:9. doi: 10.1186/s12900-017-0079-7 (PMC5738077; doi:10.1186/s12900-017-0079-7)

## Slide 1
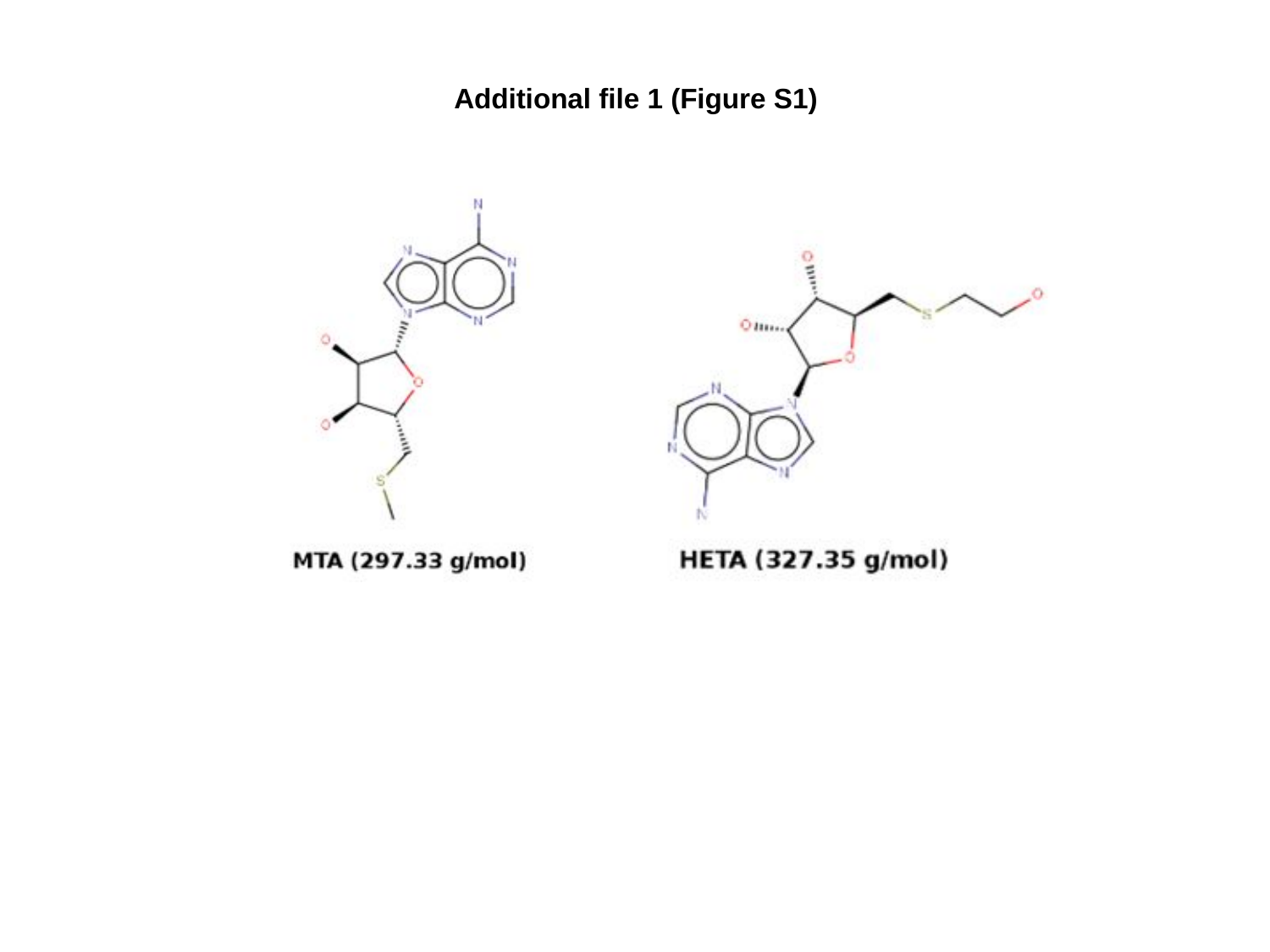

Additional file 1 (Figure S1)

Supplement: Supplementary file 1 — Chemical structure of MTA and HETA, docked in HuMTAP, LiMTAP and TbMTAP active sites. (PPTX 66 kb) [file 12900_2017_79_MOESM1_ESM.pptx]

## Slide 1
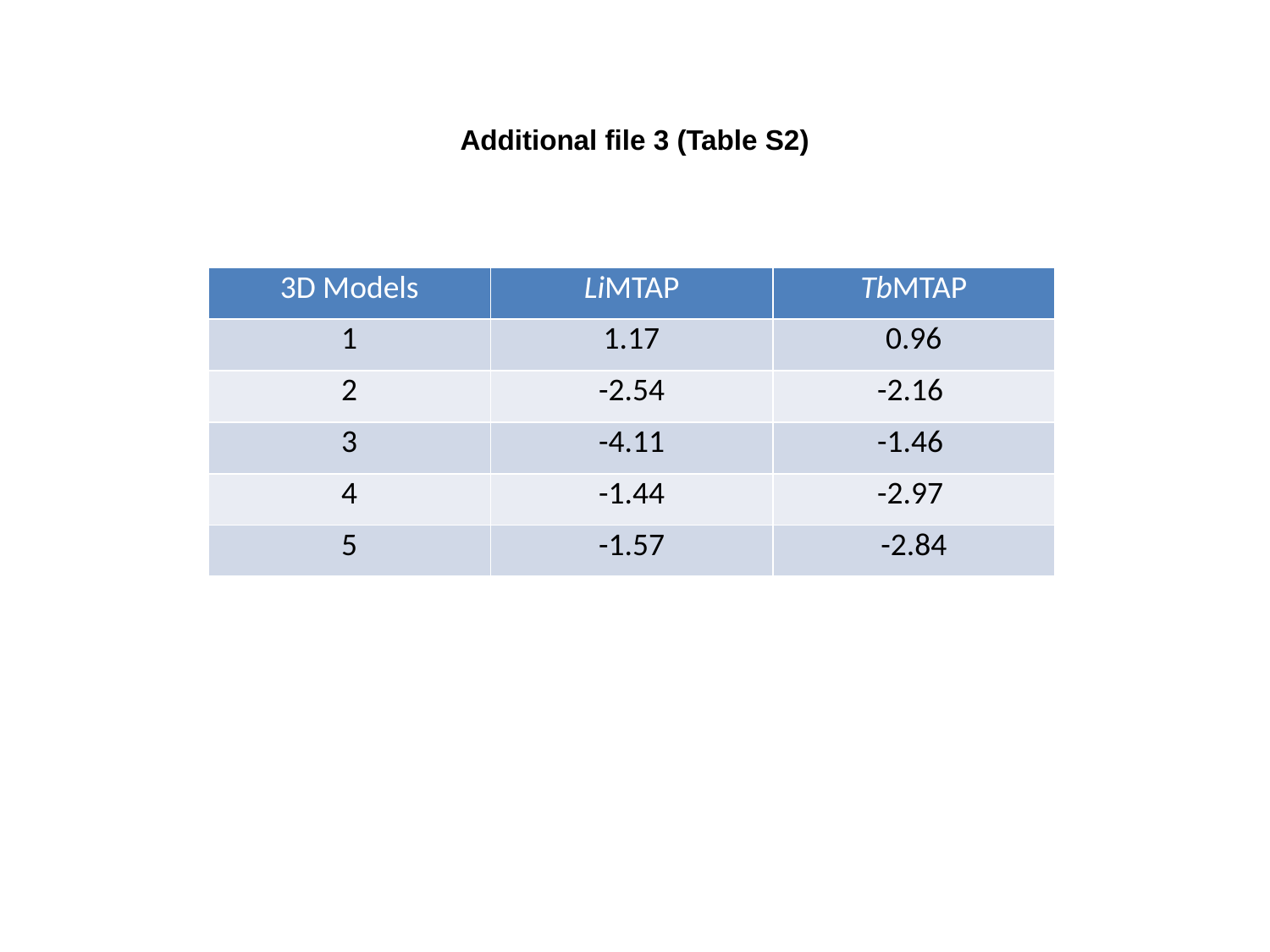

# Additional file 3 (Table S2)
| 3D Models | LiMTAP | TbMTAP |
| --- | --- | --- |
| 1 | 1.17 | 0.96 |
| 2 | -2.54 | -2.16 |
| 3 | -4.11 | -1.46 |
| 4 | -1.44 | -2.97 |
| 5 | -1.57 | -2.84 |

Supplement: Supplementary file 3 — C-scores of the five models generated by I-TASSER for LiMTAP and TbMTAP. The first model was retained for each protein (LiMTAP and TbMTAP) as it presented the highest C-score. (PPTX 44 kb) [file 12900_2017_79_MOESM3_ESM.pptx]

## Slide 1
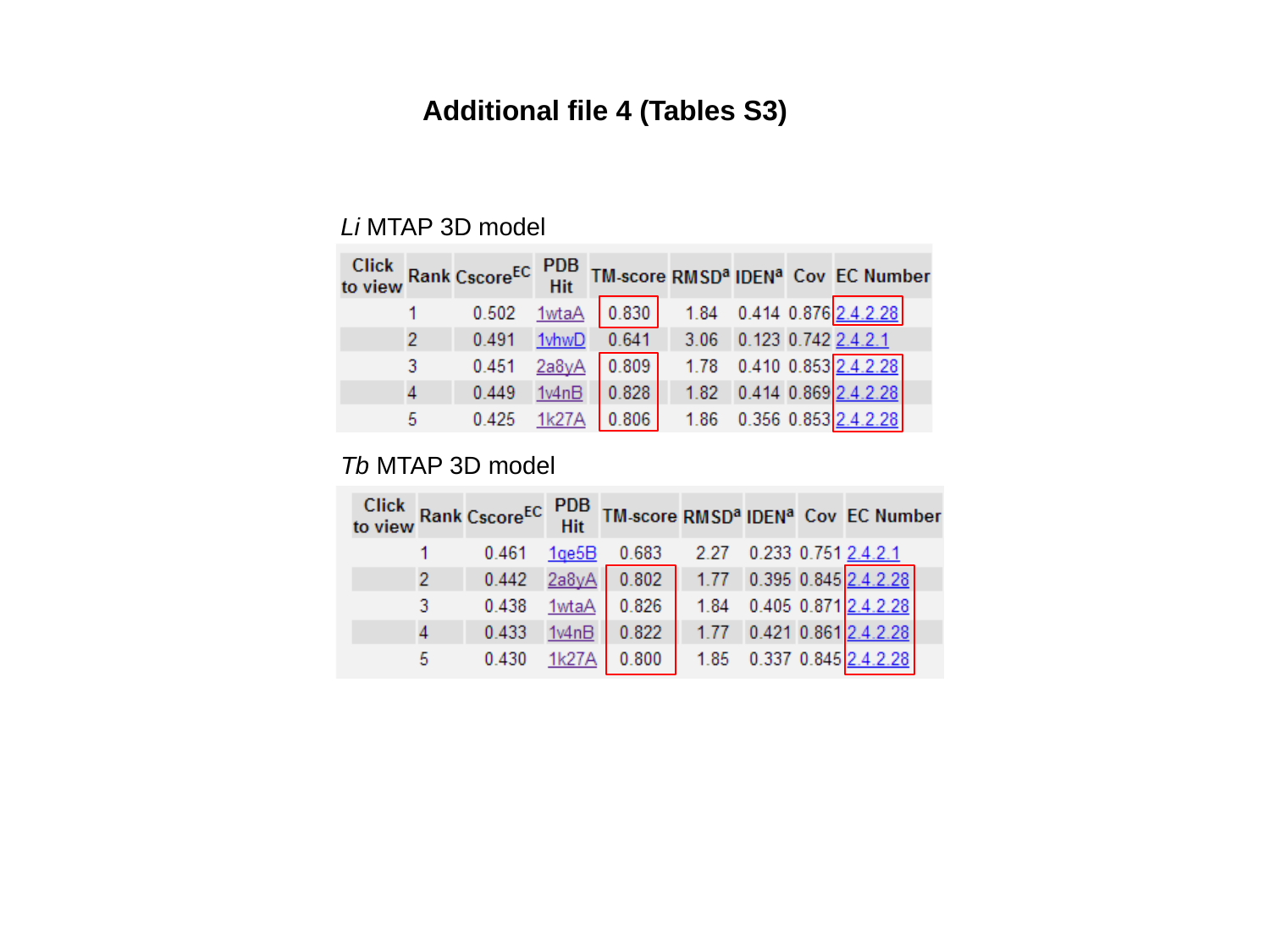

# Additional file 4 (Tables S3)
Li MTAP 3D model
Tb MTAP 3D model

Supplement: Supplementary file 4 — EC number predictions provided by I-TASSER for LiMTAP and TbMTAP 3D models. Four hits among the five returned had the highest TM-scores and 2.4.2.28 as EC number, which corresponds to 5’-Methylthioadenosine phosphorylase. (PPTX 86 kb) [file 12900_2017_79_MOESM4_ESM.pptx]

## Slide 1
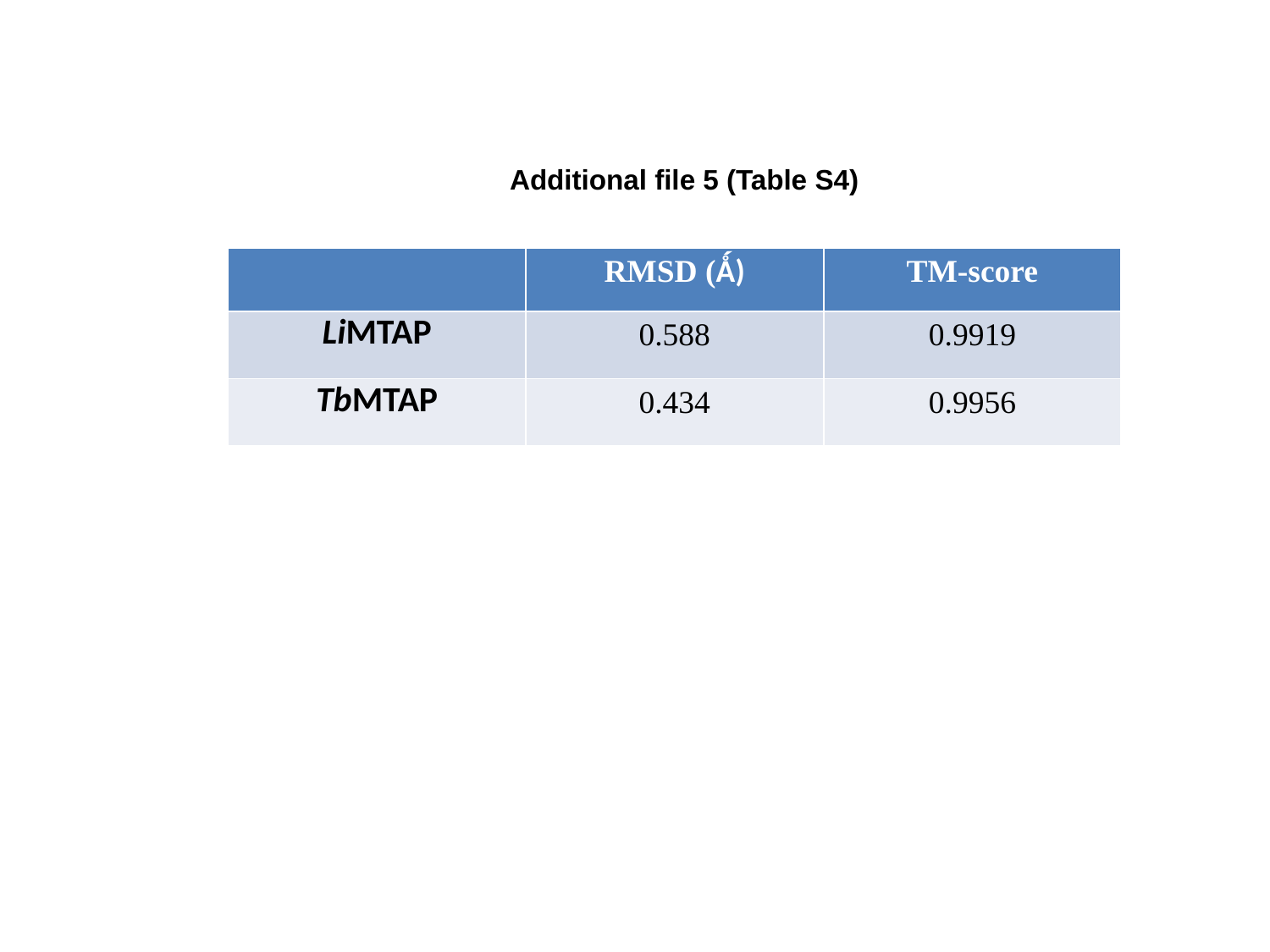

# Additional file 5 (Table S4)
| | RMSD (Ǻ) | TM-score |
| --- | --- | --- |
| LiMTAP | 0.588 | 0.9919 |
| TbMTAP | 0.434 | 0.9956 |

Supplement: Supplementary file 5 — RMSD (Ǻ) and TM-score of the refined 3D models of LiMTAP and TbMTAP. Refined models returned low RMSD values and high TM-scores. (PPTX 39 kb) [file 12900_2017_79_MOESM5_ESM.pptx]

## Slide 1
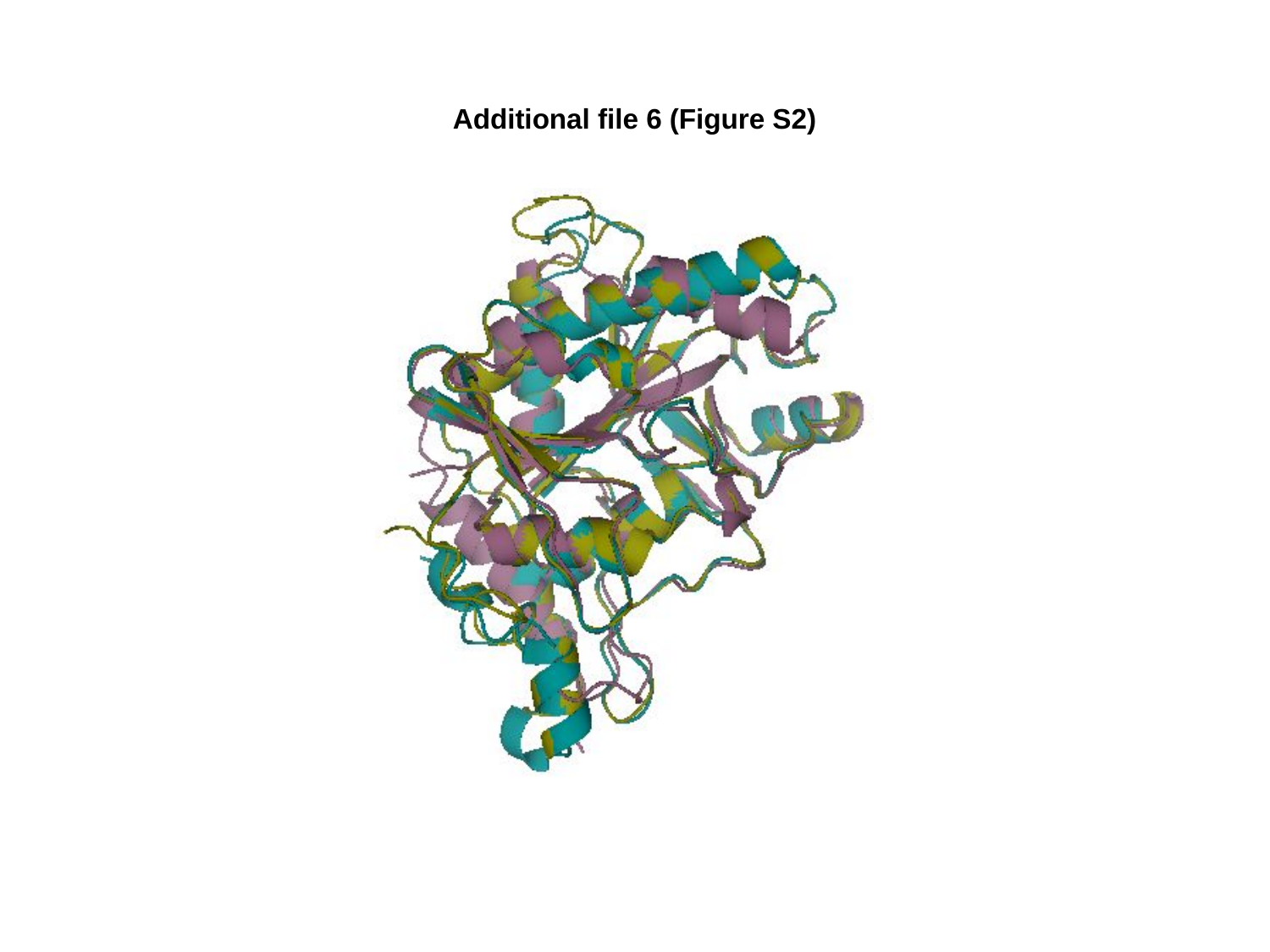

# Additional file 6 (Figure S2)

Supplement: Supplementary file 6 — Alignment of LiMTAP and TbMTAP 3D models on the Human crystal structure (PDB: 1CG6). HuMTAP, LiMTAP and TbMTAP were represented by cartoons and colored in violet, cyan and yellow, respectively. The three MTAP models aligned perfectly. (PPTX 151 kb) [file 12900_2017_79_MOESM6_ESM.pptx]

## Slide 1
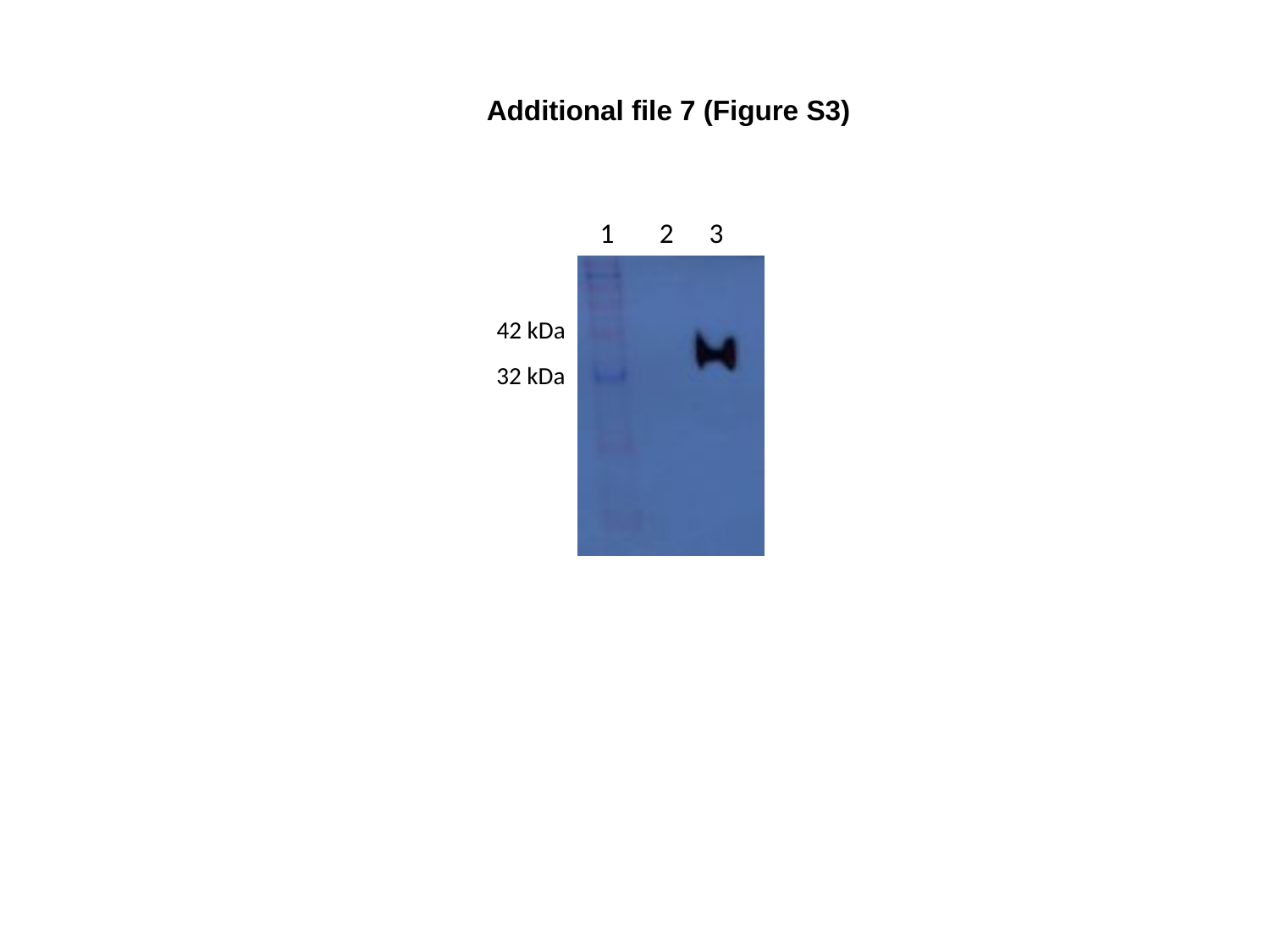

Additional file 7 (Figure S3)
1
2
3
42 kDa
32 kDa

Supplement: Supplementary file 7 — Characterization of the polyclonal antibody directed against an antigenic C-terminal peptide of LiMTAP. The total proteins extracted from L. infantum and human THP1 cells were resolved on 12% SDS-PAGE gel, transferred to PVDF membrane and then subjected to western blot analysis using anti-LiMTAP (1/10000) antibody. The Figure is representative of three independent experiments. Lanes: (1) Prestained marker MW in kDa (Vivantis, CA, USA); (2) THP1 lysates; (3) Fifteen micrograms of L. infantum (LV50) promastigote lysates. (PPTX 45 kb) [file 12900_2017_79_MOESM7_ESM.pptx]
