# Supplementary material for: Leishmania infantum 5’-Methylthioadenosine Phosphorylase presents relevant structural divergence to constitute a potential drug target
Source: BMC Struct Biol. 2017 Dec 19;17:9. doi: 10.1186/s12900-017-0079-7 (PMC5738077; doi:10.1186/s12900-017-0079-7)
Supplement: Supplementary file 2 — Taxonomy blast reports for MEME motifs. Organism, blast name, score, number of hits and organism description were provided for each MEME motif report. (a) M5 motif, (b) M7 motif, (c) M4 motif, (d) M8 motif, (e) M6 motif. (PPTX 974 kb) [file 12900_2017_79_MOESM2_ESM.pptx]

## Slide 1
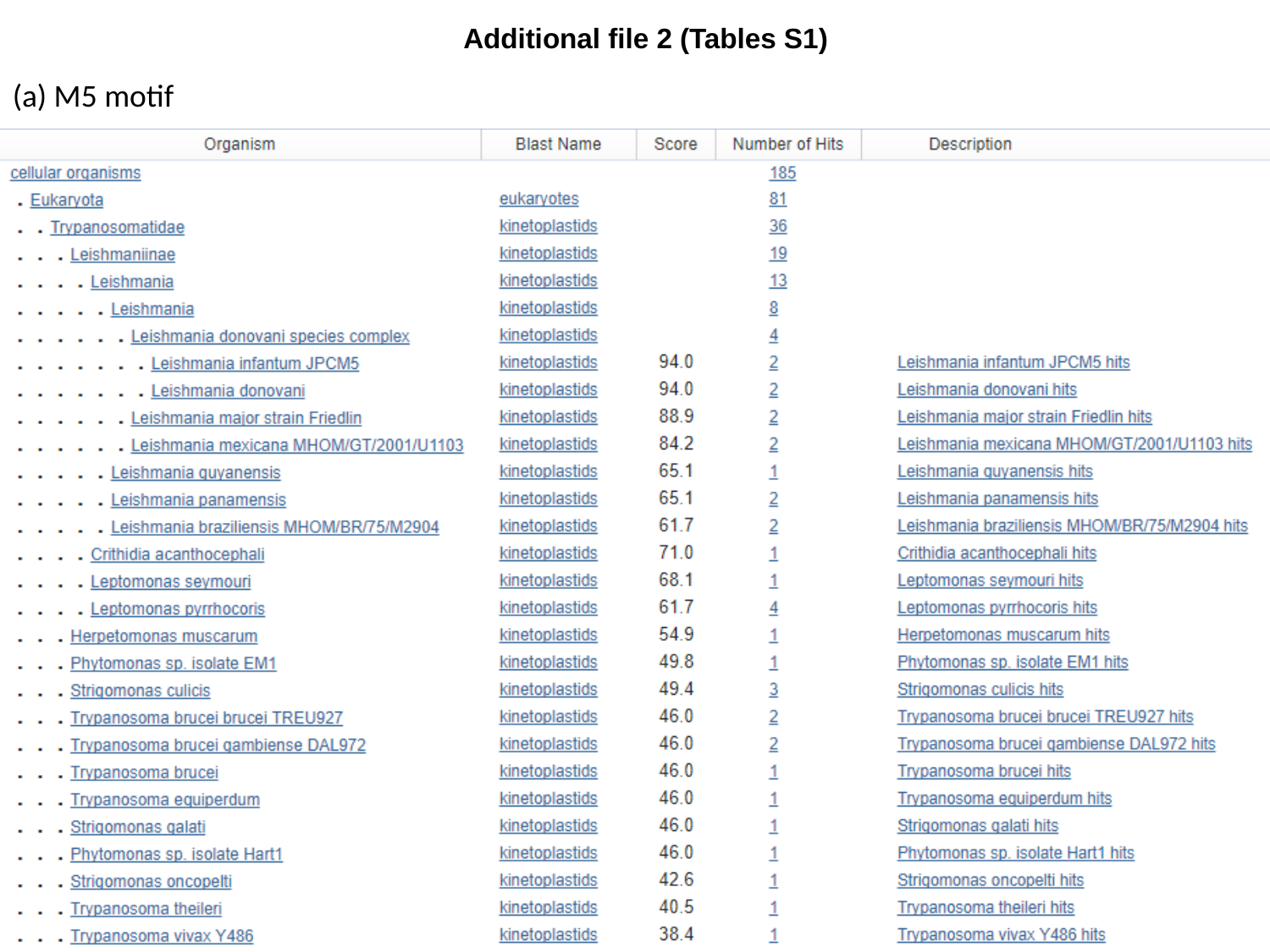

Additional file 2 (Tables S1)
(a) M5 motif
#

## Slide 2
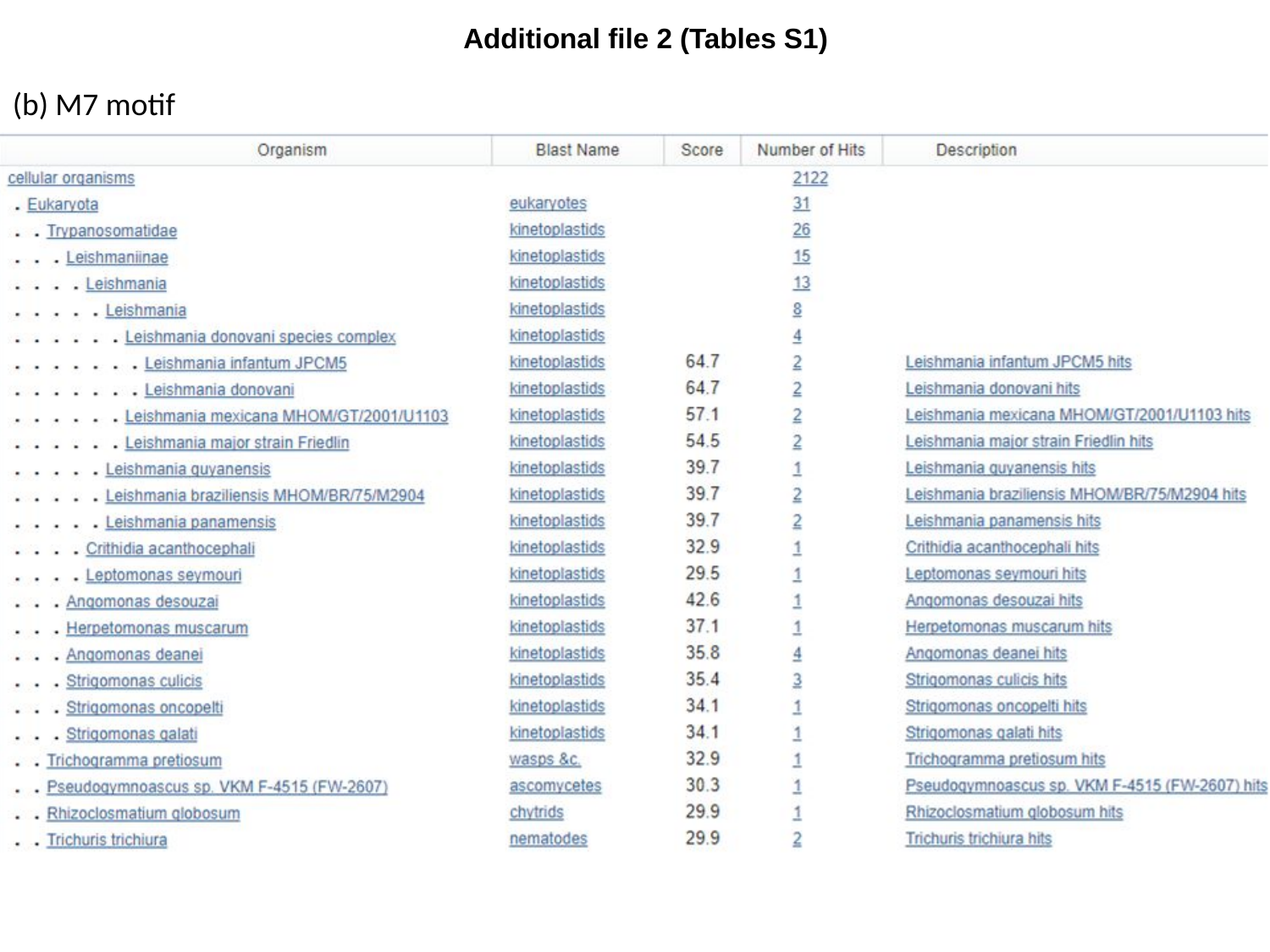

Additional file 2 (Tables S1)
(b) M7 motif

## Slide 3
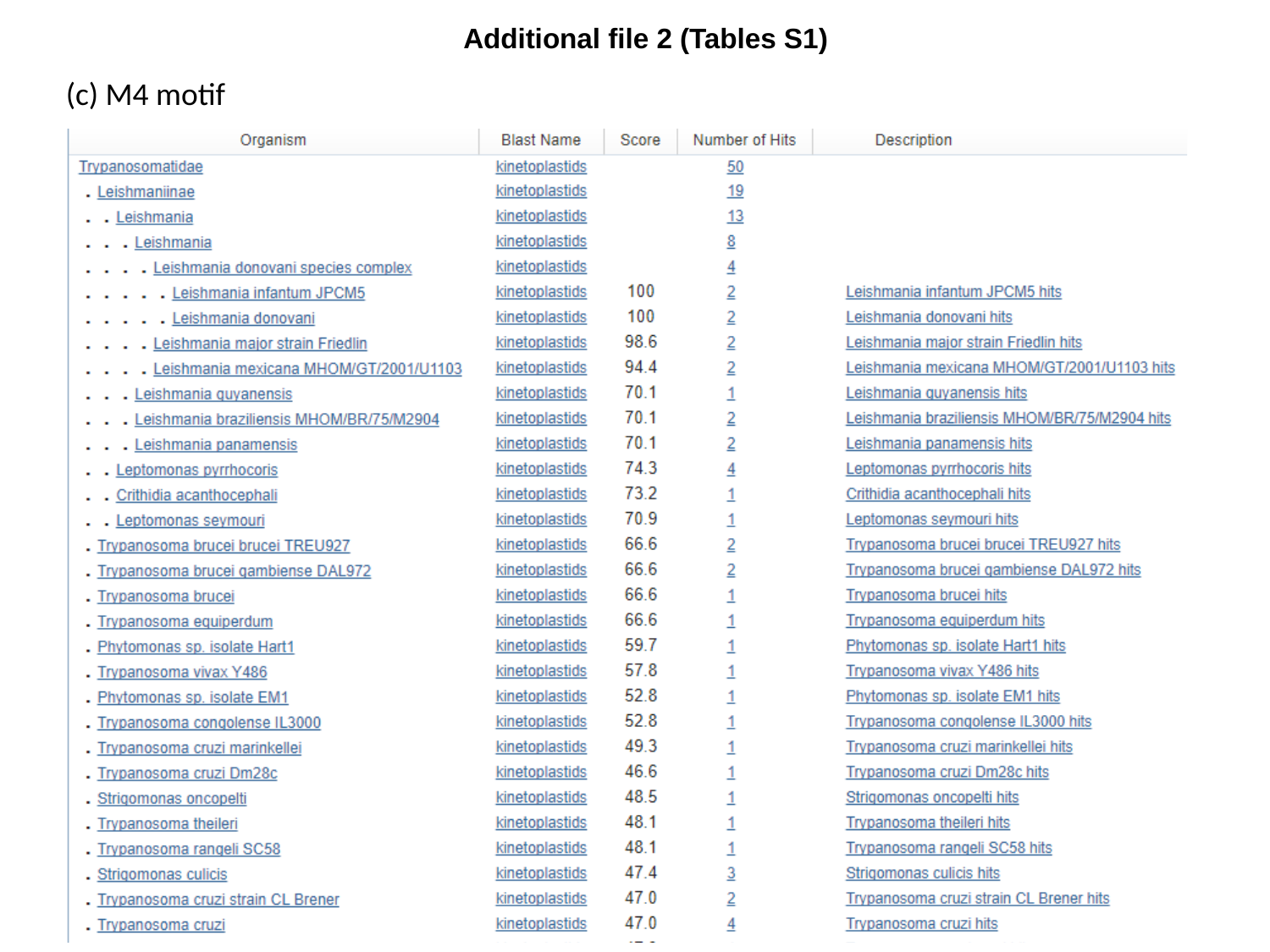

Additional file 2 (Tables S1)
(c) M4 motif

## Slide 4
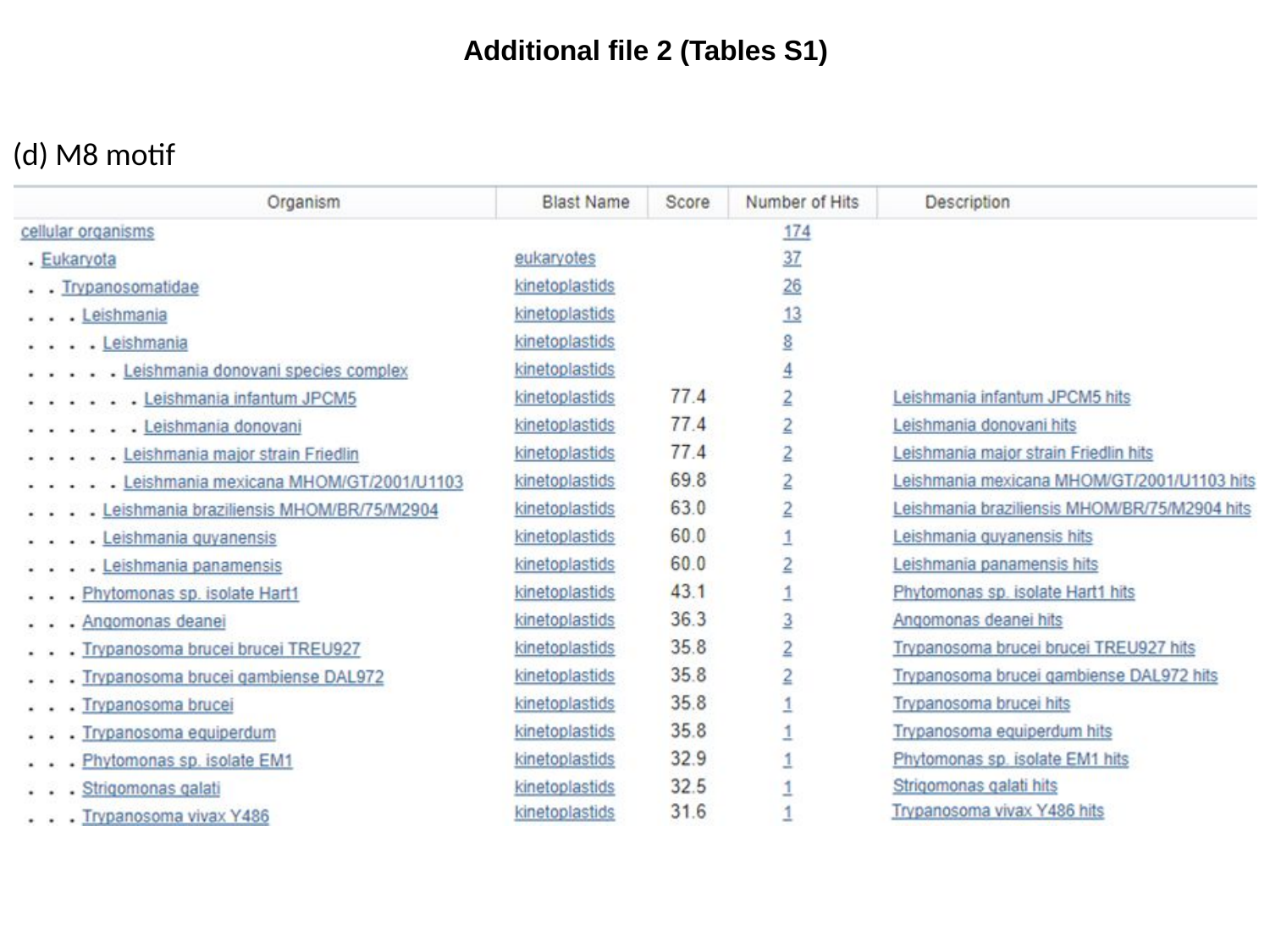

Additional file 2 (Tables S1)
(d) M8 motif

## Slide 5
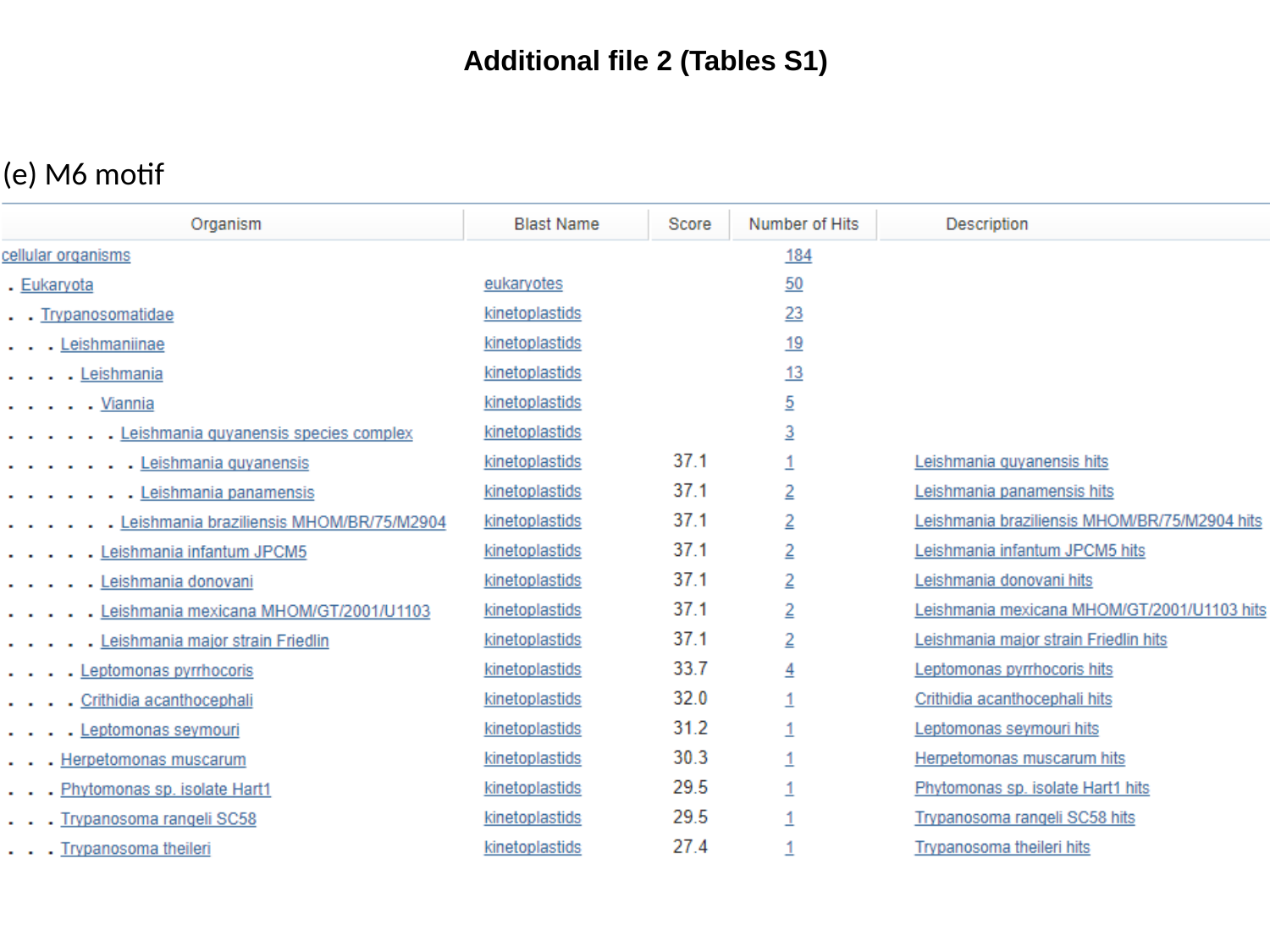

Additional file 2 (Tables S1)
(e) M6 motif
